# Supplementary material for: Moral distress measurement in animal care workers: a systematic review
Source: BMJ Open. 2024 Apr 19;14(4):e082235. doi: 10.1136/bmjopen-2023-082235 (PMC11033641; doi:10.1136/bmjopen-2023-082235)
Supplement: Author's manuscript [file bmjopen-2023-082235.draft_revisions.pdf]

BMJ Open

BMJ Open is committed to open peer review. As part of this commitment we make the peer review history of every article we publish publicly available.

When an article is published we post the peer reviewers' comments and the authors' responses online. We also post the versions of the paper that were used during peer review. These are the versions that the peer review comments apply to.

The versions of the paper that follow are the versions that were submitted during the peer review process. They are not the versions of record or the final published versions. They should not be cited or distributed as the published version of this manuscript.

BMJ Open is an open access journal and the full, final, typeset and author-corrected version of record of the manuscript is available on our site with no access controls, subscription charges or pay-per-view fees (http://bmjopen.bmj.com).

If you have any questions on BMJ Open's open peer review process please email info.bmjopen@bmj.com

Moral distress measurement in animal care workers: a systematic review

Journal:	*BMJ Open*
Manuscript ID	bmjopen-2023-082235
Article Type:	Original research
Date Submitted by the Author:	17-Nov-2023
Complete List of Authors:	Goy, Nastassja; University of Lausanne, Department of occupational and environmental health<br>Baysal, Yigit; University of Lausanne, Department of occupational and environmental health<br>Hartnack, Sonja; University of Zurich, Secion of Epidemiology, Vetsuisse Faculty<br>Canu, Irina ; Unisanté, Department of occupational and environmental health
Keywords:	Behavior, Psychological Stress < Stress, Psychological, Psychometrics, Occupational Stress, ETHICS (see Medical Ethics)

SCHOLARONE™
Manuscripts

BMJ

I, the Submitting Author has the right to grant and does grant on behalf of all authors of the Work (as defined in the below author licence), an exclusive licence and/or a non-exclusive licence for contributions from authors who are: i) UK Crown employees; ii) where BMJ has agreed a CC-BY licence shall apply, and/or iii) in accordance with the terms applicable for US Federal Government officers or employees acting as part of their official duties; on a worldwide, perpetual, irrevocable, royalty-free basis to BMJ Publishing Group Ltd ("BMJ") its licensees and where the relevant Journal is co-owned by BMJ to the co-owners of the Journal, to publish the Work in this journal and any other BMJ products and to exploit all rights, as set out in our licence.

The Submitting Author accepts and understands that any supply made under these terms is made by BMJ to the Submitting Author unless you are acting as an employee on behalf of your employer or a postgraduate student of an affiliated institution which is paying any applicable article publishing charge ("APC") for Open Access articles. Where the Submitting Author wishes to make the Work available on an Open Access basis (and intends to pay the relevant APC), the terms of reuse of such Open Access shall be governed by a Creative Commons licence – details of these licences and which Creative Commons licence will apply to this Work are set out in our licence referred to above.

Other than as permitted in any relevant BMJ Author's Self Archiving Policies, I confirm this Work has not been accepted for publication elsewhere, is not being considered for publication elsewhere and does not duplicate material already published. I confirm all authors consent to publication of this Work and authorise the granting of this licence.

For peer review only

Moral distress measurement in animal care workers: a systematic review

Nastassja Goy[1*], Yigit Baysal[2], Sonja Hartnack[2], Irina Guseva Canu[1]

[1]Department of occupational and environmental health, Unisanté, University of Lausanne, Lausanne, Switzerland

[2]Section of Epidemiology, Vetsuisse Faculty, University of Zurich, Zurich, Switzerland

*Corresponding author. Email: yigit.baysal@uzh.ch; Address: Section of Epidemiology, Vetsuisse Faculty, University of Zurich, Winterthurerstrasse 270, CH-8057, Zürich, Switzerland

For peer review only

For peer review only - http://bmjopen.bmj.com/site/about/guidelines.xhtml

Abstract

Background: The mental health of veterinary professionals is significantly impacted by the psychological stressors they encounter, such as euthanasia, witnessing animal suffering, and moral distress. Moral distress, initially identified in nursing, arises when individuals are aware of the right action but are hindered by institutional constraints. There is no consensus on assessing moral distress in animal care workers, crucial for developing preventive strategies. We aimed to review existing research on moral distress among animal care workers, by focusing on the identification and psychometric validity of its measurement.

Methods: The review followed the two-step systematic review protocol (CRD42023422259). First, we identified all moral distress measures used in animal care research, in the eligible original studies extracted from PubMed, EMBASE, and PsycINFO. Then, we evaluated their psychometric validity, emphasizing content validity, which is a critical aspect of patient-reported outcome measures (PROMs). This evaluation adhered to the Consensus-based Standards for the Selection of Health Measurement Instruments (COSMIN). The results were reported according to the Preferred Reporting Items for Systematic Reviews and Meta-Analysis and COSMIN guidelines.

Results: The review identified only one PROM specifically adapted for veterinary contexts: the Measure of Moral Distress for Animal Professionals (MMD-AP), derived from the Measure of Moral Distress for Healthcare Professionals (MMD-HP). Both MMD-HP and MMD-AP were evaluated for the quality of development and content validity. The development quality of both measures was deemed doubtful. MMD-HP's content validity was rated as sufficient, whereas MMD-AP's was inconsistent. However, the evidence quality for both PROMs was rated low.

Discussion: This systematic review, the first focusing on measuring moral distress in animal care workers, reveals a significant gap in standardized, evidence-based methods for assessing moral distress in this field.

For peer review only

The findings underscore the need for developing and validating dedicated measures tailored to the unique context of animal care.

Funding: This project has received funding from Swiss National Science Foundation (SNF) for the NRP79 project 407940_206396 “Linking animal and human welfare – refining rodent euthanasia”.

Protocol registration: The study protocol was preregistered in Prospective Register of Systematic Reviews (PROSPERO) under the registration number (CRD42023422259).

Keywords: Ethics; content validity; psychometric properties; patient-reported outcome measures (PROMs); occupational health.

Strengths and Limitations

- The adherence to COSMIN guidelines for assessing the content validity of PROMs ensures a rigorous evaluation of the measures of moral distress (MD) and their psychometric validity.
- The study followed a well-defined two-step systematic review process, adhering to the PRISMA guidelines and registering with PROSPERO. This ensures a thorough and transparent methodological approach.
- By synthesizing robust assessment of COSMIN framework with transparent methodological approach, this study primes the design and standardization of valid MD assessments.
- The strength of the review's conclusions is inherently dependent on the quality and robustness of the original studies included.
- Moral distress has been coined in 1984 in the nursing discipline and since then further definitions have been developed. It is possible that researchers focused on our target populations used other terms to describe the phenomenon of MD, and we therefore, missed publications.

Summary Box:

For peer review only

What is already known on this topic?

- Scientific knowledge on moral distress (MD) primarily focused on healthcare workers, few data on other occupations.
- MD is defined as knowing the right action but being constrained from taking it. MD affects both personal and professional quality of life. MD can lead to or exacerbate mental ill-heath.

What this study adds?

- Among 560 articles initially identified, only one presented a standardized measurement of MD for animal care professionals (MMD-AP).
- MMD-AP has several development and validity issues that need addressing. A previous measure designed for healthcare professionals (MMD-HP) demonstrates more robust psychometric properties.

How this study might affect research, practice policy?

- Further validation and improvement of available measures of MD in animal care workers are necessary.

For peer review only - http://bmjopen.bmj.com/site/about/guidelines.xhtml

Background

Rationale

The World Health Organization (WHO) defines mental health as a state of well-being essential for coping with daily life and engaging in meaningful work, beyond just the absence of mental disorders (1). The Global Burden of Disease (GBD) study highlights a bidirectional relationship between work and mental health (2). Participating in meaningful work serves as a protective factor for mental health, while mental well-being influences occupational functioning and quality of professional life (1). However, work-related stressors, particularly those with moral challenges, can negatively impact mental health (3). Occupational mental health issues like burnout, compassion fatigue, and moral distress (MD) have been studied in healthcare workers but less so in other professions facing similar stressors, such as animal care workers (4,5,6). Animal care workers, including lab technicians and veterinarians, are at risk of mental ill-health and suicide, particularly due to unique stressors like euthanasia (4,5,7,8,9). Compared to human healthcare professionals, research on moral distress among animal care workers is limited, though it also potentially impacts animal welfare and the implementation of the 3R principles (replacement, reduction, refinement). A significant study showed an association between poorer quality of life in laboratory animal personnel and limited control over euthanasia procedures (10).

Moral distress was first described in nursing as "when one knows the right thing to do, but institutional constraints make it nearly impossible to pursue the right course of action" (11, 12). It involves complicity in wrongdoing, lack of voice, and conflict with professional values, often exacerbated in hierarchical work environments (12, 13). In veterinary medicine, moral distress arises from practices like euthanasia and witnessing animal suffering, with personal experiences and attitudes towards animals influencing its development. Veterinarians also report high levels of compassion fatigue, anxiety, burnout, depression, and suicidal thoughts (7,8,9). Moral distress affects both professional and personal quality of life and can exacerbate PTSD, burnout, and compassion fatigue (9, 14, 15).

For peer review only - http://bmjopen.bmj.com/site/about/guidelines.xhtml

Despite several systematic reviews on moral distress in medical workers, none have specifically addressed animal care workers. Various tools, mostly self-administered questionnaires, or Patient-Reported Outcome Measures (PROMs) (16) have been developed to measure moral distress. Front of such diversity, a question of their relevance and validity in the context of animal care practice arises.

Objectives

In this study, we aimed to systematically review the available literature to answer two complementary research questions: 1-Which PROMs are used in animal workers to assess moral distress? and 2-What is the psychometric quality of these PROMs? The quality of a PROM is defined by its validity, reliability, and responsiveness, with the content validity being the most important psychometric property (17). According to the COnsensus-based Standards for the selection of health Measurement INstruments (COSMIN), the content validity is "the degree to which the content of an instrument is an adequate reflection of the construct to be measured" (17). Thus, we focused on the content validity, while assessing the quality of the identified moral distress measures.

Methods

The study involved two steps: initially, a systematic review to identify moral distress measures used in animal care worker studies. By measure, we understand any standardized questionnaire or scale, some of which could be a PROM. PROMs are validated, standardized, and self-reported screening tools (i.e., questionnaires/ rating scales) for patients' subjective assessments of their quality of life, exposures, and health (18), with their validity encompassing content, face, criterion, and construct validity. The second step focused on assessing the psychometric validity of these measures, especially their content validity, as per COSMIN guidelines. This assessment involved appraising PROM development quality, content validity studies, and the relevance, comprehensiveness, and comprehensibility of the PROM content, concluding with grading the content validity and evidence quality (9). The results were reported according to the Preferred Reporting Items for Systematic Reviews and Meta-Analysis and COSMIN guidelines (19)

For peer review only - http://bmjopen.bmj.com/site/about/guidelines.xhtml

Step 1. Systematic review of the moral distress measurements used in animal care workers

The study protocol was conducted according to protocol registered in Prospective Register of Systematic Reviews (PROSPERO) under the registration number (CRD42023422259).

Eligibility criteria

In this review we included the studies meeting the following criteria: 1-original (primary) study conducted in animal care workers, describing either 2-the development of a moral distress measure, or 3-validation of a moral distress measure in its original or modified version, to assess at least one of the psychometric properties mentioned in COSMIN guidelines (i.e., content validity, face validity, construct validity, structural validity hypothesis testing, cross-cultural validity, criterion validity, internal consistency, reliability, measurement error, responsiveness, interpretability) (20), 4-published in English, German, French, Turkish, or Russian languages. We excluded: 1-reviews, editorials, commentaries, and conference papers, 2-studies using or assessing other outcomes than moral distress, and 3-grey literature.

Data sources and search terms

We used three databases: PubMed, EMBASE, and PsycINFO to search for eligible studies published between January 1988 and April 2023. An experienced librarian developed the research strategy that consisted of free-text words to define three search strings, the terms focusing on the population of interest (i.e., animal care workers), terms related to moral distress, and terms related to PROMs. The latter was facilitated using the application of a sensitive filter developed by Terwee et al. (17). Reference lists were manually checked to identify additional studies.

Study screening and selection

The librarian imported all items retrieved from each of the three databases into the bibliography software EndNote 20 and removed the duplicates. The items were then imported into the Rayyan application (21)

For peer review only - http://bmjopen.bmj.com/site/about/guidelines.xhtml

for screening. The screening of eligible studies was conducted by two independent reviewers in two steps. The first screening was conducted based on the title and abstract according to the criteria described above. All eligible or unclassifiable items were included in the second screening, conducted based on the full-text reading. In both steps, reviewers discussed the discrepancies, and when necessary, they consulted a third reviewer. As part of the quality control, 25% of the items in both steps of screening were cross-checked by a fourth independent reviewer with expertise in veterinary research.

Step 2. Systematic review of psychometric validity of the identified moral distress measures

This review was conducted according to the COSMIN guidelines for systematic reviews of PROMs (20) and the practical guidelines on the identification of the most valid PROM (22). First, we searched in the COSMIN database for the existent studies of moral distress PROM development or systematic reviews of their validation (17). We identified a systematic review by Gianetta et al. (23), which included 32 moral distress measures in original, revised, and translated versions. Although the authors stated that their review was conducted according to COSMIN guidelines, the content validity of the available moral distress PROMs was not adequately assessed. Consequently, we conducted the assessment of the content validity of the PROMs identified in step 1. Content validity involves the relevance of the items which constitute the PROM, their comprehensiveness, and comprehensibility by the target population. Content validity depends on the quality of the PROM development and the pilot studies of its validation, which should include qualitative and quantitative methods (17).

The COSMIN manuals provide well-defined criteria for each step of the assessment and recommend that each PROM and PROM dimension shall be assessed separately and by two independent reviewers. We strictly followed all COSMIN recommendations and used the assessment tools and tables for result reporting provided in the COSMIN manual (17). At the end of each step, the reviewers' appraisal results were discussed in the presence of a third reviewer to get a consensus for each property.

For peer review only - http://bmjopen.bmj.com/site/about/guidelines.xhtml

Data extraction and synthesis

We considered the following information relevant for extraction: study reference, name and reference of the moral distress measure used, psychometric properties assessed, and methods and results of their assessments. Whenever necessary the reviewers contacted the study authors to complete this information. The collected information was then summarized in a narrative synthesis.

Patient and Public Involvement Statement

Patients or the public were not involved in the design, or conduct, or reporting, or dissemination plans of our research.

Results

Included studies

The literature search resulted in 560 items. After duplicates removal, 455 records were retained for the first screening based on titles and abstracts. For the second screening based on the full-text screening, we selected 16 articles, from which only one met the inclusion criteria (Figure 1). In the other articles, either the moral distress was not measured (n=13), or it was an ineligible type of publication: such as a dissertation (n=1), or the article did not satisfy the inclusion criteria (n=1), only using the moral distress prevalence as one of the covariates.

The included study was published in 2023 by Kogan et al. (13) and aimed to assess moral distress in veterinarians. Kogan et al. found that gender and age were predictors of moral distress. Indeed, the level of moral distress was higher in young women than in older men. Moreover, the authors found that a high level of moral distress was associated with a low level of professional fulfillment and with a high level of exhaustion, burnout, and interpersonal disengagement (13).

For peer review only - http://bmjopen.bmj.com/site/about/guidelines.xhtml

Identified measures of moral distress

Kogan et al. (13) used a modified version of the Measure of Moral Distress for Health Professionals (MMD-HP) that for the sake of clarity, we recalled the Measure of Moral Distress for Animal Professionals (MMD-AP). The MMD-HP was a PROM originally developed by Epstein et al. (24). Kogan and al. (13) revised the MMD-HP by creating two PROM versions, one for owners and one for associates to suit the veterinary context. For example, the authors added items related to customers' financial resources and whether they can afford treatment for their animals. These kinds of items were not present in the MMD-HP.

In the COSMIN framework, all revised versions of a PROM are considered as new PROMs and should be assessed independently. Therefore, in the second step of this study, we considered both PROMs: MMD-HP and MMD-AP for associates. We chose this version because we think it makes sense to have, at first, a PROM for most of veterinarians. There are more often employed veterinarians than veterinarians who have their own veterinary practice. Moreover, the associates' version is the same as the owners' version except that there are three more items. These items are related to superiors' pressure and their lack of support. Thus, we think this version is more representative of the animal workers' job experience and covers all items in the both MMD-AP versions.

Construct definition and PROM description

The two identified PROMs share the same construct of Moral distress, initially defined by Jameton (11). Epstein et al. (24), defined it later as follows: *"The pressure to act unethically is the defining concept of the phenomenon and it separates those situations that are emotionally distressing or otherwise morally troubling from those that threaten moral integrity"*. Epstein et al. (24) considered five important factors as causes of moral distress that inspired the MMD-HP structure: complicity in wrongdoing, lack of voice, professional values, the repetitive nature of moral distress, and the three roots of causes. These three roots were ordered at three different levels: patients or their families, for example when the family asks for a

For peer review only - http://bmjopen.bmj.com/site/about/guidelines.xhtml

treatment that does more harm to the patient than good. The colleagues or team can be a cause of moral distress, for example in the case of poor communication between team members. Finally, the system can cause moral distress when there is a lack of human, material, or financial resources. Moral distress can be a combination of stressors arising at these three levels. For instance, if the worker faces pressure situations in which he/she cannot report an error committed by a colleague frequently, his/her opinion cannot be considered and his/her professional ethic could be violated (24).

Measure of Moral Distress for Health Professionals (MMD-HP)

The first identified PROM, MMD-HP was developed in 2019, by revising the Moral Distress Scale Revised (MDS-R) (25). Epstein et al. revised MDS-R to make it applicable to all health workers. MDS-R itself originally stems from the Moral Distress Scale (MDS) (26). Epstein et al. underwent the construction of a new PROM to overlook the drawbacks of the original and previous revised versions, namely to simplify the use and better integrate the causal levels of moral distress, more specifically, the team and system levels. Epstein et al. reviewed the literature over the past five years to identify new roots of causes of moral distress. The new elements led to the deletion of three items, the modification of three other items, the consolidation of two items, and the addition of 11 new items. The new items integrated the team and system levels, which according to Epstein et al., were a weakness of MDS-R, mostly focused on the patient level. The MMD-HP contains 27 items and the three levels of roots to separate the items into four factors. Some items appear in more than one factor. The first factor corresponds to the primarily system level, while the second factor corresponds to the clinical level at the patient level. Factors 3 and 4 both represent the team level. However, factor 3 contains items, which imply a threat to personal integrity from a member of his team, whereas factor 4 concerns the team's communication with patients and families.

The MMD-HP intends a double scaling, one for the frequency of the described situations and one for the degree of distress each situation results in. Both are scored on a 5-point Likert scale, ranging from 0 (never) to 4 (very frequently) for the frequency and from 0 (none) to 4 (very distressing) for the degree of distress. The product of the frequency and the distress scores corresponds to an overall score of moral distress,

For peer review only - http://bmjopen.bmj.com/site/about/guidelines.xhtml

ranging from 0 to 432. The higher the score, the greater the level of moral distress. However, the authors did not mention any cut-off values (17).

The second PROM, MMD-AP resulted from an additional revision of the MMD-HP. Kogan et al. (13) used the MMD-HP (24) to assess moral distress in veterinarians. The associates' version, for employed veterinarians, includes 24 items, and the owners' version has 21 items. The authors explained that they removed items, which did not fit the veterinary context. The authors considered the five components described above, which were included in the MMD-AP. For example, for the system level, one item reads: "*Experience lack of administrative action or support for a problem that is compromising patient care"*. For the patient level, an item reads: *"Euthanasia based on client's unwillingness to treat"*. For the team level, there is an item: *"Feel pressured by superiors to order or carry out orders for what I consider to be unnecessary or inappropriate tests and treatments"*. For each item, the response scale is based on the frequency and the distress experienced. Response options and calculation of the moral distress overall score are the same as the MMD-HP.

Quality of psychometric validity of the selected PROMs

Quality of the PROM development

The COSMIN guidelines for evaluating the quality of the study on the PROM development consist of two parts. The first part deals with the standards used to assess the quality of the searches carried out to find the most suitable items for the new PROM. It includes general design requirements and concept elicitation. The concept of elicitation is important for relevance and comprehensiveness of the PROM. The second part looks at the standards used to assess the quality of the cognitive interview study or pilot studies conducted. The aim is to rate the comprehensiveness and comprehensibility of the PROM (17).

We assessed the quality of development of MMD-HP and MMD-AP. We also contacted the authors for further information on the analyses they carried out.

For peer review only - http://bmjopen.bmj.com/site/about/guidelines.xhtml

For both PROMs, the construct and its origin, the target population, and the context of use were well-defined and rated as very good. Both PROMs have been developed in English and tested in a sample representing the target population, thus the general design requirements were also rated as very good. We appraised the concept elicitation as doubtful for MMD-AP and adequate for MMD-HP. For these two PROMs, we could not rate them as very good, because the qualitative data collection was not or not enough described. The total PROM design was thus rated as adequate for MMD-HP and doubtful for MMD-AP. The MMD-AP authors conducted a very good cognitive interview asking the target population (i.e., veterinarians) for guidance and feedback, thus we evaluated the general design requirements as very good. For MMD-HP, the rating was adequate because the cognitive interview was performed with experts and clinicians but not with all health professionals. However, Kogan et al. did not ask the participants about the comprehensibility of the items included in the PROM. Comprehensibility is the standard that ensures that PROM is comprehensible to the target population. For a very good rating, different PROM components need to be evaluated separately: instruction, item, response option, and recall period (17). Since the authors did not mention whether they have evaluated these components for MMD-AP, we rated the quality of the comprehensibility study as doubtful. Finally, the total quality of the MMD-AP development study was rated as doubtful. For MMD-HP, the comprehensibility was also assessed as doubtful. Although its authors carried out a study to evaluate the face validity, they did not mention whether they used an interview guide and did verbatim transcriptions.

Table 1 summarizes the results of the PROM development assessment, with the items considered and their consensual rating by two independent reviewers.

Quality of the PROM content validity

The criteria to assess content validity are relevance, comprehensiveness, and comprehensibility. The relevance means, for example, ensuring that PROM items are adapted to the target population and to the context in which they are used. The comprehensiveness evaluates the completeness of the PROM and the

For peer review only

comprehensibility concerns the understanding of the PROM by the target population. Table 2 summarizes the results of these three criteria for PROMs content validity assessment.

For the MMD-AP content validity assessment, the development study was evaluated as inconsistent. The lack of information on qualitative data collection for the development of this PROM led to an insufficient assessment of the relevance. For comprehensiveness, the key concepts were missing, and we rated it as insufficient. The comprehensibility was also rated as insufficient because the authors did not report whether they tested or not the understanding of the PROM. Both reviewers rated the development study of this PROM as sufficient. The reviewers estimated that the PROM itself contained the necessary information. Consequently, the overall rating of the MMD-AP was inconsistent. There was no content validity study available for this PROM (17). Therefore, the lack of studies on content validity and the doubtful PROM development study has resulted in a low quality of evidence.

The content validity of MMD-HP was rated as sufficient. Indeed, the relevance, comprehensiveness, and comprehensibility were rated as sufficient, and the two reviewers also evaluated these properties as sufficient. Overall, the content validity rating was also sufficient. Since we did not find any content validity study for MMD-HP, the quality of evidence grading started as moderate. Then, we downgraded it to low because the PROM's development study has been assessed as doubtful.

Table 3 summarizes the results of the PROMs content validity assessment, with the items considered and their consensual rating by two independent reviewers.

Other psychometric properties of the selected PROMs

According to the COSMIN framework, in case the PROM does not meet the criteria for a sufficient quality of content validity, the assessment of other psychometric proprieties is useless, the content validity being a paramount requirement for recommending a PROM for use in research or clinical practice. The content validity was inconsistent for MDD-AP and sufficient for MDD-HP, therefore, we did not conduct any formal assessment of the other properties of these PROMs. Nevertheless, Gianetta et al. made a systematic

For peer review only - http://bmjopen.bmj.com/site/about/guidelines.xhtml

review of studies assessing additional psychometric properties of MDD-HP (23). The internal consistency of MDD-HP was graded as sufficient, with a Cronbach alpha of 0.93 on average (23). The structural validity was appraised as sufficient. It was assessed by an exploratory factor analysis, where the four identified factors accounted for 54.3% of the variance (23). However, any confirmatory factor analysis was conducted, conversely to COSMIN guidelines for the structural validity quality (16). Gianetta et al. evaluated the cross-cultural validity and hypothesis testing as sufficient. The reliability was appraised as sufficient with the inter-rater agreement of 88% (23). Gianetta et al. didn't report any analysis concerning measurement error, responsiveness, and construct validity of MMD-HP. Finally, they rated the criterion validity as insufficient. Indeed, there is no gold standard test of moral distress enabling the assessment of this property.

Discussion

This study focused on identifying valid Patient-Reported Outcome Measures (PROMs) to assess moral distress in animal care workers. A two-step systematic review revealed the scarcity of standardized measures in this field. Only one specific measure for veterinarians, the Measure of Moral Distress for Animal Professionals (MMD-AP), was identified and evaluated for content validity following COSMIN guidelines (13, 16). This evaluation found the quality of MMD-AP development to be doubtful, with inconsistent content validity and low-quality evidence (17).

The findings highlight several critical issues. Firstly, moral distress in animal care workers has not received adequate attention compared to other outcomes like anxiety, compassion fatigue, or burnout. Secondly, many studies claiming to measure moral distress did not follow a valid or standardized methodology. Thirdly, there seems to be a disregard for proper PROM development and psychometric validation methods in current research in this area. This gap in methodological rigor hinders understanding the nature, determinants, and prevention of moral distress among animal care workers. However, this issue is not unique to animal care but is also prevalent in other fields, as demonstrated by the review of Gianetta et al

For peer review only - http://bmjopen.bmj.com/site/about/guidelines.xhtml

(23). Their review, included in the COSMIN database, did not assess the content validity of the reviewed PROMs as required by COSMIN guidelines, reflecting a broader trend of methodological shortcomings in current research practices.

The only PROM available for measuring moral distress in animal care workers is the MMD-AP (13). Due to its current inconsistent content validity and low-quality evidence, further studies are necessary for its validation and improvement. Before its reassessment, the MMD-AP is not recommended for use in research or clinical practice, as it fails to meet COSMIN criteria of relevance, comprehensiveness, and comprehensibility.

Improving the quality of MMD-AP's content validity assessment requires qualitative studies involving the target population, including animal shelter workers, lab workers, and veterinary nurses. This may lead to adaptations or new item additions, resulting in a revised MMD-AP version, which would require its validation studies.

An accurate assessment of a health problem in terms of incidence or prevalence requests a validated standard for measuring this problem in clinical, occupational, or research settings. It could ideally be a validated diagnostic test, or a consensually approved set of diagnostic criteria, such as those used for quantifying the burden of major depressive disorder or general anxiety disorder in the global burden of diseases (27). Unless such tests or criteria have been established, PROMs can help estimate the amount of the problem, as it was recently shown in occupational burnout research (22, 28). Yet, the prerequisite for this is an appropriate choice of the most valid PROM (29, 30). Compared to occupational burnout, moral distress is a relatively recent construct in occupational psychology, therefore, the research on moral distress could benefit from and capitalize on the methodological development in subjective outcome measurement in the other fields.

Conclusion

For peer review only - http://bmjopen.bmj.com/site/about/guidelines.xhtml

This is the first systematic review focused on moral distress measurement in animal care workers. It showed that moral distress is rarely measured using standardized and evidence-based methods. These findings underline the need to develop and thoroughly validate such methods in the context of animal care. We think it is also necessary for the well-being of the animals, as we believe that moral distress has an impact on the animals cared for by the animal caretakers. workers. More generally, methodological improvement and training in psychometry are recommended when developing and validating measures of moral distress and other subjective outcomes.

Figure Legends

Figure 1. Flow-chart of study selection

Declarations

- **Word count:** 3999

- **Ethics approval and consent to participate**

  Not applicable.

- **Consent for publication**

  Not applicable.

- **Availability of data and materials**

  Data are available online as supplemental material of the present article.

- **Competing interests**

  None declared.

For peer review only - http://bmjopen.bmj.com/site/about/guidelines.xhtml

385 • **Funding**

386 This project has received funding from Swiss National Science Foundation (SNF) for the NRP79
387 project 407940_206396 “Linking animal and human welfare – refining rodent euthanasia”.

388 • **Authors' contributions**

389 Conceptualization, I.G.C.; methodology, N.G., Y.B., and I.G.C.; software, N.G. and Y.B.;
390 validation, N.G., Y.B., I.G.C. and S.H; formal analysis, N.G., and Y.B.; investigation, N.G., Y.B.,
391 and I.G.C.; resources, N.G., Y.B., and I.G.C.; data curation, N.G., Y.B., and I.G.C.; writing—
392 original draft preparation, N.B., Y.B., and I.G.C; writing—review and editing, N.G., Y.B., I.G.C.
393 and S.H.; supervision, I.G.C. and S.H ; project administration, I.G.C. and S.H; funding acquisition,
394 I.G.C and S.H. All authors have read and agreed to the published version of the manuscript.

395 • **Acknowledgements**

396 The authors thank the Unisanté documentarist, Thomas Brauchly, for his precious help in
397 establishing the literature search queries.

References

1. World Health Organization. WHO guidelines on mental health at work. Geneva: World Health Organization; 2022.
2. Wang H, Abbas KM, Abbasifard M, Abbasi-Kangevari M, Abbastabar H, Abd-Allah F, et al. Global age-sex-specific fertility, mortality, healthy life expectancy (HALE), and population estimates in 204 countries and territories, 1950–2019: a comprehensive demographic analysis for the Global Burden of Disease Study 2019. Lancet. 2020;396(10258):1160-203.
3. Crane MF, Phillips JK, Karin E. Trait perfectionism strengthens the negative effects of moral stressors occurring in veterinary practice. Aust Vet J. 2015;93(10):354-60.

For peer review only - http://bmjopen.bmj.com/site/about/guidelines.xhtml

4. Shoman Y, Rousson V, Bianchi R, Guseva Canu I. Holistic Assessment of Factors Associated with Exhaustion, the Main Symptom of Burnout: A Meta-Analysis of Longitudinal Studies. Int J Environ Res Public Health. 2022;19(20).

5. Al-Gobari M, Shoman Y, Blanc S, Canu IG. Point prevalence of burnout in Switzerland: a systematic review and meta-analysis. Swiss Med Wkly. 2022;152:w30229.

6. Burdorf A. How to improve intervention research on the psychosocial work environment? Scand J Work Environ Health. 2023;49(5):311-3.

7. Guseva Canu I, Bovio N, Mediouni Z, Bochud M, Wild P. Suicide mortality follow-up of the Swiss National Cohort (1990-2014): sex-specific risk estimates by occupational socio-economic group in working-age population. Soc Psychiatry Psychiatr Epidemiol. 2019;54(12):1483-95.

8. Moses L, Malowney MJ, Wesley Boyd J. Ethical conflict and moral distress in veterinary practice: A survey of North American veterinarians. J Vet Intern Med. 2018;32(6):2115-22.

9. Arbe Montoya AI, Hazel S, Matthew SM, McArthur ML. Moral distress in veterinarians. Vet Rec. 2019;185(20):631.

10. LaFollette MR, Riley MC, Cloutier S, Brady CM, O'Haire ME, Gaskill BN. Laboratory Animal Welfare Meets Human Welfare: A Cross-Sectional Study of Professional Quality of Life, Including Compassion Fatigue, Among Laboratory Animal Personnel. Qual Life Res. 2018;27(5):1147-57.

11. Jameton A. Dilemmas of moral distress: moral responsibility and nursing practice. AWHONNS Clin Issues Perinat Womens Health Nurs. 1993;4(4):542-51.

12. Jameton A, Jackson EM. Nuclear war and nursing ethics. What is the nurse's responsibility? Möbius: J Contin Educ Prof Health Sci. 1984;4(1):75-88.

13. Kogan LR, Rishniw M. Veterinarians and moral distress. J Am Vet Med Assoc. 2023;261(5):1-7.

14. Reeve C, Rogelberg S, Spitzmueller C, Digiacomo N. The Caring-Killing Paradox: Euthanasia-Related Strain Among Animal-Shelter Workers. J Appl Soc Psychol. 2006;35:119-43.

15. Andrukonis A, Hall NJ, Protopopova A. The Impact of Caring and Killing on Physiological and Psychometric Measures of Stress in Animal Shelter Employees: A Pilot Study. Int J Environ Res Public Health. 2020;17(24).

16. Mokkink LB, de Vet HCW, Prinsen CAC, Patrick DL, Alonso J, Bouter LM, et al. COSMIN Risk of Bias checklist for systematic reviews of Patient-Reported Outcome Measures. Qual Life Res. 2018;27(5):1171-9.

17. Terwee CB, Prinsen CAC, Chiarotto A, Westerman MJ, Patrick DL, Alonso J, et al. COSMIN methodology for evaluating the content validity of patient-reported outcome measures: a Delphi study. Qual Life Res. 2018;27(5):1159-70.

18. Kingsley C, Patel S. Patient-reported outcome measures and patient-reported experience measures. BJA Educ. 2017;17(4):137-44.

19. Moher D, Liberati A, Tetzlaff J, Altman DG. Preferred reporting items for systematic reviews and meta-analyses: the PRISMA statement. PLoS Med. 2009;6(7):e1000097.

For peer review only - http://bmjopen.bmj.com/site/about/guidelines.xhtml

20. Prinsen CAC, Mokkink LB, Bouter LM, Alonso J, Patrick DL, de Vet HCW, et al. COSMIN guideline for systematic reviews of patient-reported outcome measures. Qual Life Res. 2018;27(5):1147-57.
21. Ouzzani M, Hammady H, Fedorowicz Z, Elmagarmid A. Rayyan-a web and mobile app for systematic reviews. Syst Rev. 2016;5(1):210.
22. Shoman Y, Majery N, Otelea M, Lambreghts C, Guseva Canu I. How to identify the most suitable questionnaires and rating scales for your clinical practice or research? Int J Clin Pract. 2021;75(12): e14895.
23. Giannetta N, Villa G, Pennestrì F, Sala R, Mordacci R, Manara DF. Instruments to assess moral distress among healthcare workers: A systematic review of measurement properties. Int J Nurs Stud. 2020; 111:103767.
24. Epstein EG, Whitehead PB, Prompahakul C, Thacker LR, Hamric AB. Enhancing Understanding of Moral Distress: The Measure of Moral Distress for Health Care Professionals. AJOB Empir Bioeth. 2019;10(2):113-24.
25. Hamric AB. Empirical research on moral distress: issues, challenges, and opportunities. HEC Forum. 2012;24(1):39-49.
26. Corley MC, Elswick RK, Gorman M, Clor T. Development and evaluation of a moral distress scale. J Adv Nurs. 2001;33(2):250-6.
27. Santomauro DF, Mantilla Herrera AM, Shadid J, Zheng P, Ashbaugh C, Pigott DM, et al. Global prevalence and burden of depressive and anxiety disorders in 204 countries and territories in 2020 due to the COVID-19 pandemic. Lancet. 2021;398(10312):1700-12.
28. Guseva Canu I, Marca SC, Dell'Oro F, Balázs Á, Bergamaschi E, Besse C, et al. Harmonized definition of occupational burnout: A systematic review, semantic analysis, and Delphi consensus in 29 countries. Scand J Work Environ Health. 2021;(2):95-107.
29. Guseva Canu I, Mesot O, Györkös C, Mediouni Z, Mehlum IS, Bugge MD. Burnout syndrome in Europe: towards a harmonized approach in occupational health practice and research. Ind Health. 2019;57(6):745-52.
30. Shoman Y, Marca S, Bianchi R, Godderis L, Molen HF, Guseva Canu I. Psychometric properties of burnout measures: A systematic review. Epidemiol Psychiatr Sci. 2021;30:e8.

For peer review only

Table 1. Quality of the PROM development

**PROM**	**MMD-HP**	**MMD-AP**
First author, year	Esptein et al., 2019	Kogan et al., 2023
Language in which the PROM was developed	English	English
**PROM design**		

For peer review only - http://bmjopen.bmj.com/site/about/guidelines.xhtml

Clear construct	V	V
Clear origin of construct	V	V
Clear target population for which the PROM was developed	V	V
Clear context of use	V	V
PROM developed in sample representing the target population	V	V
Concept elicitation	A	D
Total PROM design	***A***	***D***
Cognitive interview study performed in sample representing the target population	A	V
Comprehensibility	D	D
Total Cognitive interview study	***D***	***D***
**Total PROM development**	**D**	**D**

Note*: V: very good, A: adequate, D: doubtful*

Table 2. Systematic results for the MMD-HP and MMD-AP PROMs according to COSMIN

**PROM**	**MMD-HP**		**MMD-AP**	
**COSMIN psychometric**	**Overall rating + / - / ?**	**Quality of evidence High, moderate, low, very low**	**Overall rating + / - / ?**	**Quality of evidence High, moderate, low, very low**

For peer review only - http://bmjopen.bmj.com/site/about/guidelines.xhtml

properties for content validity				
Relevance	+	**Low**	±	**Low**
Comprehensiveness	+	**Low**	-	**Low**
Comprehensibility	+	**Low**	-	**Low**

Note: +*: sufficient,* -*: insufficient,* ±: *inconsistent*

For peer review only

Table 3. Systematic results for the MMD-HP and MMD-AP PROMs for the content validity according to COSMIN

PROM	Development study	Rating of reviewers	Overall rating + / - / ?	Quality of evidence
**MMD-HP**				

For peer review only - http://bmjopen.bmj.com/site/about/guidelines.xhtml

Relevance	+	±	+	**Low**
Comprehensiveness	+	+	+	**Low**
Comprehensibility	+	+	+	**Low**
**Content validity rating**	+	+	+	**Low**
**MMD-AP**				
Relevance	±	+	±	**Low**
Comprehensiveness	-	+	±	**Low**
Comprehensibility	-	+	±	**Low**
**Content validity rating**	±	+	±	**Low**

Note: +*: sufficient, -: insufficient,* ±: *inconsistent*

For peer review only

For peer review only - http://bmjopen.bmj.com/site/about/guidelines.xhtml

Table 1. Quality of the PROM development

PROM	MMD-HP	MMD-AP
First author, year	Esptein et al., 2019	Kogan et al., 2023
Language in which the PROM was developed	English	English
**PROM design**		
Clear construct	V	V
Clear origin of construct	V	V
Clear target population for which the PROM was developed	V	V
Clear context of use	V	V
PROM developed in sample representing the target population	V	V
Concept elicitation	A	D
Total PROM design	***A***	***D***
Cognitive interview study performed in sample representing the target population	A	V
Comprehensibility	D	D
Total Cognitive interview study	***D***	***D***
**Total PROM development**	**D**	**D**

Note*: V: very good, A: adequate, D: doubtful*

For peer review only

Table 2. Systematic results for the MMD-HP and MMD-AP PROMs according to COSMIN

**PROM**	**MMD-HP**		**MMD-AP**	
**COSMIN psychometric properties for content validity**	**Overall rating**<br>**+ / - / ?**	**Quality of evidence**<br>**High, moderate, low, very low**	**Overall rating**<br>**+ / - / ?**	**Quality of evidence**<br>**High, moderate, low, very low**
Relevance	+	**Low**	±	**Low**
Comprehensiveness	+	**Low**	-	**Low**
Comprehensibility	+	**Low**	-	**Low**

Note: +*: sufficient, -: insufficient,* ±: *inconsistent*

For peer review only

Table 3. Systematic results for the MMD-HP and MMD-AP PROMs for the content validity according to COSMIN

PROM	Development study	Rating of reviewers	Overall rating<br>+ / - / ?	Quality of evidence
**MMD-HP**				
Relevance	+	±	+	**Low**
Comprehensiveness	+	+	+	**Low**
Comprehensibility	+	+	+	**Low**
**Content validity rating**	+	+	+	**Low**
**MMD-AP**				
Relevance	±	+	±	**Low**
Comprehensiveness	-	+	±	**Low**
Comprehensibility	-	+	±	**Low**
**Content validity rating**	±	+	±	**Low**

Note: +*: sufficient, -: insufficient,* ±: *inconsistent*

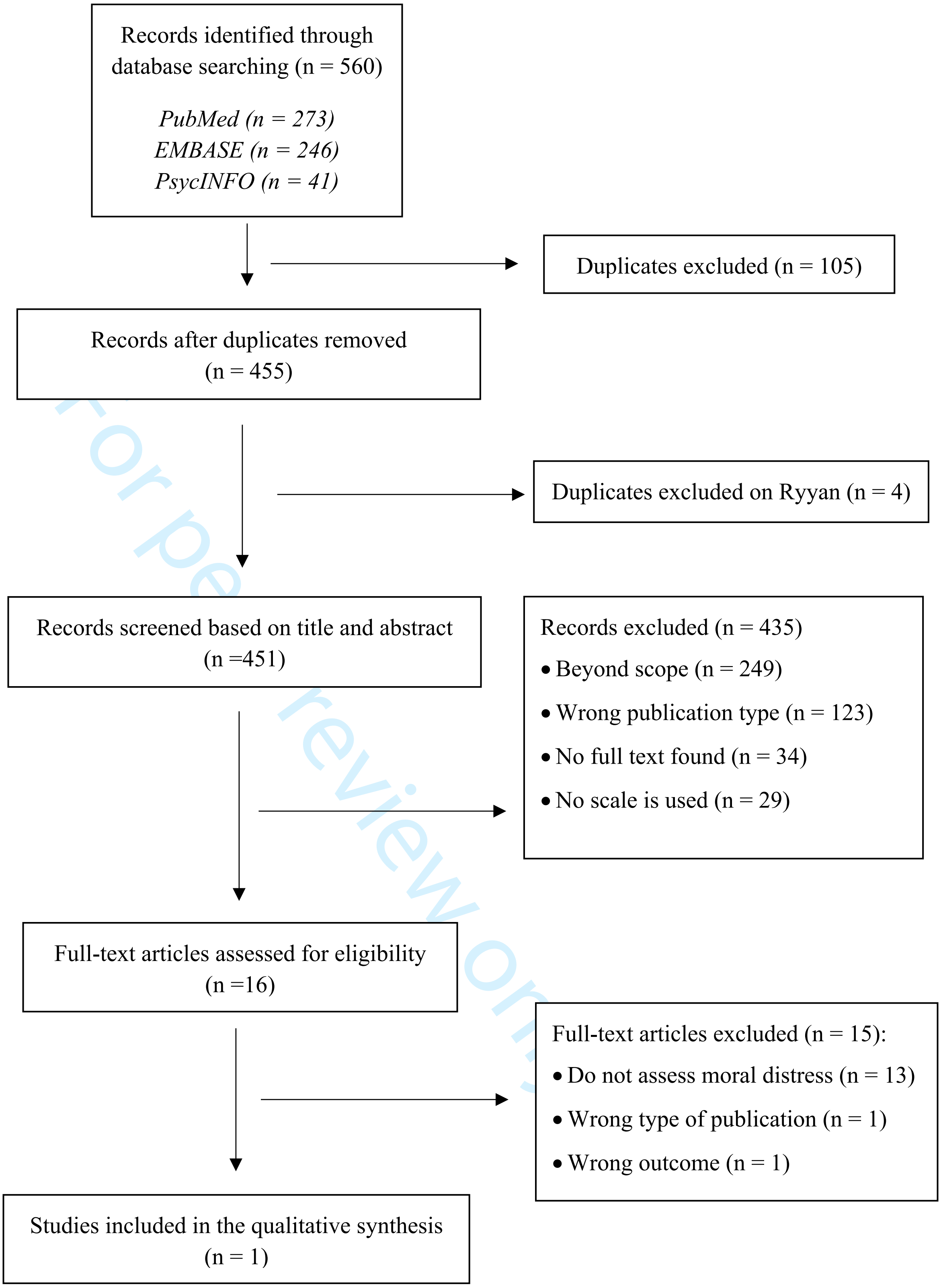

Figure 1: Flow-chart

PRISMA 2020 for Abstracts Checklist

Section and Topic	Item #	Checklist item	Reported (Yes/No)
**TITLE**			
Title	1	Identify the report as a systematic review.	Yes
**BACKGROUND**			
Objectives	2	Provide an explicit statement of the main objective(s) or question(s) the review addresses.	Yes
**METHODS**			
Eligibility criteria	3	Specify the inclusion and exclusion criteria for the review.	Yes
Information sources	4	Specify the information sources (e.g. databases, registers) used to identify studies and the date when each was last searched.	Yes
Risk of bias	5	Specify the methods used to assess risk of bias in the included studies.	Yes
Synthesis of results	6	Specify the methods used to present and synthesise results.	Yes
**RESULTS**			
Included studies	7	Give the total number of included studies and participants and summarise relevant characteristics of studies.	Yes
Synthesis of results	8	Present results for main outcomes, preferably indicating the number of included studies and participants for each. If meta-analysis was done, report the summary estimate and confidence/credible interval. If comparing groups, indicate the direction of the effect (i.e. which group is favoured).	Yes
**DISCUSSION**			
Limitations of evidence	9	Provide a brief summary of the limitations of the evidence included in the review (e.g. study risk of bias, inconsistency and imprecision).	Yes
Interpretation	10	Provide a general interpretation of the results and important implications.	Yes
**OTHER**			
Funding	11	Specify the primary source of funding for the review.	Yes
Registration	12	Provide the register name and registration number.	Yes

From: Page MJ, McKenzie JE, Bossuyt PM, Boutron I, Hoffmann TC, Mulrow CD, et al. The PRISMA 2020 statement: an updated guideline for reporting systematic reviews. BMJ 2021;372:n71. doi: 10.1136/bmj.n71

For more information, visit: http://www.prisma-statement.org/

For peer review only

PRISMA 2020 Checklist

Section and Topic	Item #	Checklist item	Location where item is reported
**TITLE**			
Title	1	Identify the report as a systematic review.	1
**ABSTRACT**			
Abstract	2	See the PRISMA 2020 for Abstracts checklist.	21
**INTRODUCTION**			
Rationale	3	Describe the rationale for the review in the context of existing knowledge.	85
Objectives	4	Provide an explicit statement of the objective(s) or question(s) the review addresses.	112
**METHODS**			
Eligibility criteria	5	Specify the inclusion and exclusion criteria for the review and how studies were grouped for the syntheses.	135
Information sources	6	Specify all databases, registers, websites, organisations, reference lists and other sources searched or consulted to identify studies. Specify the date when each source was last searched or consulted.	144
Search strategy	7	Present the full search strategies for all databases, registers and websites, including any filters and limits used.	Supplemental materials
Selection process	8	Specify the methods used to decide whether a study met the inclusion criteria of the review, including how many reviewers screened each record and each report retrieved, whether they worked independently, and if applicable, details of automation tools used in the process.	151
Data collection process	9	Specify the methods used to collect data from reports, including how many reviewers collected data from each report, whether they worked independently, any processes for obtaining or confirming data from study investigators, and if applicable, details of automation tools used in the process.	160
Data items	10a	List and define all outcomes for which data were sought. Specify whether all results that were compatible with each outcome domain in each study were sought (e.g. for all measures, time points, analyses), and if not, the methods used to decide which results to collect.	167
	10b	List and define all other variables for which data were sought (e.g. participant and intervention characteristics, funding sources). Describe any assumptions made about any missing or unclear information.	Not applicable
Study risk of bias assessment	11	Specify the methods used to assess risk of bias in the included studies, including details of the tool(s) used, how many reviewers assessed each study and whether they worked independently, and if applicable, details of automation tools used in the process.	158
Effect measures	12	Specify for each outcome the effect measure(s) (e.g. risk ratio, mean difference) used in the synthesis or presentation of results.	Tables 1,2 and 3
Synthesis methods	13a	Describe the processes used to decide which studies were eligible for each synthesis (e.g. tabulating the study intervention characteristics and comparing against the planned groups for each synthesis (item #5)).	172
	13b	Describe any methods required to prepare the data for presentation or synthesis, such as handling of missing summary statistics, or data conversions.	Not applicable
	13c	Describe any methods used to tabulate or visually display results of individual studies and syntheses.	Not applicable
	13d	Describe any methods used to synthesize results and provide a rationale for the choice(s). If meta-analysis was performed, describe the model(s), method(s) to identify the presence and extent of statistical heterogeneity, and software package(s) used.	Not applicable
	13e	Describe any methods used to explore possible causes of heterogeneity among study results (e.g. subgroup analysis, meta-regression).	Not applicable
	13f	Describe any sensitivity analyses conducted to assess robustness of the synthesized results.	Not

For peer review only - http://bmjopen.bmj.com/site/about/guidelines.xhtml

PRISMA 2020 Checklist

Section and Topic	Item #	Checklist item	Location where item is reported
			applicable
Reporting bias assessment	14	Describe any methods used to assess risk of bias due to missing results in a synthesis (arising from reporting biases).	Not applicable
Certainty assessment	15	Describe any methods used to assess certainty (or confidence) in the body of evidence for an outcome.	Not applicable
**RESULTS**			
Study selection	16a	Describe the results of the search and selection process, from the number of records identified in the search to the number of studies included in the review, ideally using a flow diagram.	183
	16b	Cite studies that might appear to meet the inclusion criteria, but which were excluded, and explain why they were excluded.	186
Study characteristics	17	Cite each included study and present its characteristics.	190
Risk of bias in studies	18	Present assessments of risk of bias for each included study.	256
Results of individual studies	19	For all outcomes, present, for each study: (a) summary statistics for each group (where appropriate) and (b) an effect estimate and its precision (e.g. confidence/credible interval), ideally using structured tables or plots.	Tables 1,2 and 3
Results of syntheses	20a	For each synthesis, briefly summarise the characteristics and risk of bias among contributing studies.	310
	20b	Present results of all statistical syntheses conducted. If meta-analysis was done, present for each the summary estimate and its precision (e.g. confidence/credible interval) and measures of statistical heterogeneity. If comparing groups, describe the direction of the effect.	Not applicable
	20c	Present results of all investigations of possible causes of heterogeneity among study results.	Not applicable
	20d	Present results of all sensitivity analyses conducted to assess the robustness of the synthesized results.	Not applicable
Reporting biases	21	Present assessments of risk of bias due to missing results (arising from reporting biases) for each synthesis assessed.	Not applicable
Certainty of evidence	22	Present assessments of certainty (or confidence) in the body of evidence for each outcome assessed.	Not applicable
**DISCUSSION**			
Discussion	23a	Provide a general interpretation of the results in the context of other evidence.	331
	23b	Discuss any limitations of the evidence included in the review.	333
	23c	Discuss any limitations of the review processes used.	Not applicable
	23d	Discuss implications of the results for practice, policy, and future research.	358
**OTHER INFORMATION**			
Registration and protocol	24a	Provide registration information for the review, including register name and registration number, or state that the review was not registered.	47
	24b	Indicate where the review protocol can be accessed, or state that a protocol was not prepared.	48
	24c	Describe and explain any amendments to information provided at registration or in the protocol.	Not

For peer review only - http://bmjopen.bmj.com/site/about/guidelines.xhtml

PRISMA 2020 Checklist

Section and Topic	Item #	Checklist item	Location where item is reported
			applicable
Support	25	Describe sources of financial or non-financial support for the review, and the role of the funders or sponsors in the review.	45
Competing interests	26	Declare any competing interests of review authors.	379
Availability of data, code and other materials	27	Report which of the following are publicly available and where they can be found: template data collection forms; data extracted from included studies; data used for all analyses; analytic code; any other materials used in the review.	Supplemental materials

From: Page MJ, McKenzie JE, Bossuyt PM, Boutron I, Hoffmann TC, Mulrow CD, et al. The PRISMA 2020 statement: an updated guideline for reporting systematic reviews. BMJ 2021;372:n71. doi: 10.1136/bmj.n71

For more information, visit: http://www.prisma-statement.org/

For peer review only

Moral distress measurement in animal care workers: a systematic review

Journal:	*BMJ Open*
Manuscript ID	bmjopen-2023-082235.R1
Article Type:	Original research
Date Submitted by the Author:	15-Mar-2024
Complete List of Authors:	Baysal, Yigit; University of Zurich Vetsuisse Faculty, Section of Epidemiology<br>Goy, Nastassja; University of Lausanne, Department of occupational and environmental health<br>Hartnack, Sonja; University of Zurich Vetsuisse Faculty, Section of Epidemiology<br>Canu, Irina ; Unisanté, Department of occupational and environmental health
**Primary Subject Heading**:	Qualitative research
Secondary Subject Heading:	Public health, Occupational and environmental medicine, Nursing, Mental health
Keywords:	Behavior, Psychological Stress < Stress, Psychological, Psychometrics, Occupational Stress, ETHICS (see Medical Ethics)

SCHOLARONE™
Manuscripts

I, the Submitting Author has the right to grant and does grant on behalf of all authors of the Work (as defined in the below author licence), an exclusive licence and/or a non-exclusive licence for contributions from authors who are: i) UK Crown employees; ii) where BMJ has agreed a CC-BY licence shall apply, and/or iii) in accordance with the terms applicable for US Federal Government officers or employees acting as part of their official duties; on a worldwide, perpetual, irrevocable, royalty-free basis to BMJ Publishing Group Ltd ("BMJ") its licensees and where the relevant Journal is co-owned by BMJ to the co-owners of the Journal, to publish the Work in this journal and any other BMJ products and to exploit all rights, as set out in our licence.

The Submitting Author accepts and understands that any supply made under these terms is made by BMJ to the Submitting Author unless you are acting as an employee on behalf of your employer or a postgraduate student of an affiliated institution which is paying any applicable article publishing charge ("APC") for Open Access articles. Where the Submitting Author wishes to make the Work available on an Open Access basis (and intends to pay the relevant APC), the terms of reuse of such Open Access shall be governed by a Creative Commons licence – details of these licences and which Creative Commons licence will apply to this Work are set out in our licence referred to above.

Other than as permitted in any relevant BMJ Author's Self Archiving Policies, I confirm this Work has not been accepted for publication elsewhere, is not being considered for publication elsewhere and does not duplicate material already published. I confirm all authors consent to publication of this Work and authorise the granting of this licence.

For peer review only

Moral distress measurement in animal care workers: a systematic review

Yigit Baysal[1*, **], Nastassja Goy[2*], Sonja Hartnack[1], Irina Guseva Canu[2]

[1] Section of Epidemiology, Vetsuisse Faculty, University of Zurich, Zurich, Switzerland

[2]Department of occupational and environmental health, Unisanté, University of Lausanne, Lausanne, Switzerland

* Equally contributing authors

** Corresponding author. Email: yigit.baysal@uzh.ch; Address: Section of Epidemiology, Vetsuisse Faculty, University of Zurich, Winterthurerstrasse 270, CH-8057, Zürich, Switzerland

For peer review only - http://bmjopen.bmj.com/site/about/guidelines.xhtml

Abstract

Objectives: The mental health of veterinary and other animal health professionals is significantly impacted by the psychological stressors they encounter, such as euthanasia, witnessing animal suffering, and moral distress. Moral distress, initially identified in nursing, arises when individuals are aware of the right action but are hindered by institutional constraints. We aimed to review existing research on moral distress scales among animal care workers, by focusing on the identification and psychometric validity of its measurement.

Design: Two-step systematic review. First, we identified all moral distress scales used in animal care research, in the eligible original studies. Second, we evaluated their psychometric validity, emphasizing content validity, which is a critical aspect of patient-reported outcome measures (PROMs). This evaluation adhered to the Consensus-based Standards for the Selection of Health Measurement Instruments (COSMIN). The results were reported according to the PRISMA.

Data sources: PubMed, EMBASE, and PsycINFO to search for eligible studies published between January 1984 and April 2023.

Eligibility criteria for selecting studies: We included original (primary) studies that 1) were conducted in animal care workers; describing 2) either the development of a moral distress scale, or validation of a moral distress scale in its original or modified version, to assess at least one of the psychometric properties mentioned in COSMIN guidelines.

Data extraction and synthesis Two independent reviewers used standardized methods to search, screen and code included studies. We considered the following information relevant for extraction: study reference, name and reference of the moral distress scale used, psychometric properties assessed, and methods and results of their assessments. The collected information was then summarized in a narrative synthesis.

For peer review only

Results: The review identified only one PROM specifically adapted for veterinary contexts: the Measure of Moral Distress for Animal Professionals (MMD-AP), derived from the Measure of Moral Distress for Healthcare Professionals (MMD-HP). Both MMD-HP and MMD-AP were evaluated for the quality of development and content validity. The development quality of both measures was deemed doubtful. According to COSMIN, MMD-HP's content validity was rated as sufficient, whereas MMD-AP's was inconsistent. However, the evidence quality for both PROMs was rated low.

Conclusion: This is the first systematic review focused on moral distress measurement in animal care workers. It shows that moral distress is rarely measured using standardized and evidence-based methods and that such methods should be developed and validated in the context of animal care.

PROSPERO registration number: CRD42023422259

Funding: This project has received funding from Swiss National Science Foundation (SNF) for the NRP79 project 407940_206396 “Linking animal and human welfare – refining rodent euthanasia”.

Keywords: Ethics; content validity; psychometric properties; patient-reported outcome measures (PROMs); occupational health.

Strengths and Limitations

- The adherence to COSMIN guidelines for assessing the content validity of PROMs ensures rigorous evaluation of the scales of moral distress (MD) and their psychometric validity.
- The strength of the review's conclusions is inherently dependent on the quality and robustness of the original studies included.
- Moral distress has been coined in 1984 in the nursing discipline and since then further definitions have been developed, possibly leading to a heterogeneous and complex understanding of moral distress.

For peer review only - http://bmjopen.bmj.com/site/about/guidelines.xhtml

Background

Animal care workers' mental health and work stressors

The World Health Organization (WHO) defines mental health as a state of well-being essential for coping with daily life and engaging in meaningful work, beyond just the absence of mental disorders [1]. The Global Burden of Disease (GBD) study highlights a bidirectional relationship between work and mental health [2]. Participating in meaningful work serves as a protective factor for mental health, while mental well-being influences occupational functioning and quality of professional life [1]. However, work-related stressors, particularly those with moral challenges, can negatively impact mental health [3]. Occupational mental health issues like burnout, compassion fatigue, and moral distress (MD) have been studied in healthcare workers but less so in other professions facing similar stressors, such as animal care workers [4,5,6]. Animal care workers, including lab technicians and veterinarians, are at risk of mental ill-health and suicide, particularly due to unique stressors like euthanasia [7,8,9]. Compared to human healthcare professionals, research on moral distress among animal care workers is limited.

Both human and animal health care professionals, directly or indirectly being responsible for the care of their patients, form emotional bonds with them. However, at the same time, they may perform procedures that may cause pain and distress to their patients or find themselves unable to alleviate their pain and distress. Occupational mental health of animal care professionals has recently been emphasized; La Folleyete et al. suggested that animal care professionals are significantly experiencing emotional dissonance and moral stress [10]. A key difference between human healthcare and animal care professionals regarding workplace stress lies in the outcomes within clinical and experimental settings. The endpoint in animal experimentation is typically the death of the animal. In harm-benefit analysis it is crucially important that attempts were made to minimize harm and suffering in animal experimental setting. This ethical framework is often in conflict with predisposition of attachment, love and empathy based on human animal bonds. Animal care workers are often find themselves constantly making and breaking these emotional bonds [10].

For peer review only - http://bmjopen.bmj.com/site/about/guidelines.xhtml

Combined with the role conflict experienced due to balancing competing interest of animal welfare and human interests, moral dilemma experienced at workplace can transform into moral distress [8]. Social support networks can buffer the effects of moral dilemma and role conflict by providing emotional support and alternative perspectives that help the individual navigate ethical dilemmas and role demands. Yet, a systematic review of workplace stress in animal-care workers found reports of qualitative studies where stigma associated with animal related work tasks (e.g., euthanasia, use of animals in research) can hinder the access of social support networks [11]. Considering the complexity of moral distress in animal care work, we emphasize the need for exploration of this psychological syndrome targeted to animal care workers.

Moreover, experiencing moral distress and its negative consequences potentially impacts animal welfare, including the implementation of the 3R principle (replacement, reduction, refinement) in the animal experimental setting with animal care takers being responsible for fostering these [12]. An association between poorer quality of life in laboratory animal personnel and limited control over euthanasia procedures was found [10].

Concept of moral distress and its attributes

Moral distress was first described in nursing as "when one knows the right thing to do, but institutional constraints make it nearly impossible to pursue the right course of action" [13, 14]. It involves complicity in wrongdoing, lack of voice, and conflict with professional values, often exacerbated in hierarchical work environments [13, 14, 15]. In veterinary medicine, moral distress arises from practices like euthanasia and witnessing animal suffering, with personal experiences and attitudes towards animals influencing its development. Veterinarians also report high levels of compassion fatigue, anxiety, burnout, depression, and suicidal thoughts [7,8,9]. Moral distress affects both professional and personal quality of life and can exacerbate PTSD, burnout, and compassion fatigue [9, 16, 17].

For peer review only - http://bmjopen.bmj.com/site/about/guidelines.xhtml

Despite several systematic reviews on moral distress in medical workers, none have specifically addressed animal care workers [18]. Various tools, mostly self-administered questionnaires, or Patient-Reported Outcome Measures (PROMs) [19] have been developed to measure moral distress. Front of such diversity, a question of their relevance and validity in the context of animal care practice arises.

Study objectives

In this study, we aimed to systematically review the available literature to answer two complementary research questions: 1-Which scales or PROMs are used in animal health care workers to assess moral distress? and 2-What is the psychometric quality of these PROMs? The quality of a PROM is defined by its validity, reliability, and responsiveness, with the content validity being the most important psychometric property [19]. According to the COnsensus-based Standards for the selection of health Measurement INstruments (COSMIN) [19, 20, 21], the content validity is "the degree to which the content of a scale is an adequate reflection of the construct to be measured" [20]. Thus, we focused on the content validity, while assessing the quality of the identified moral distress measures.

Methods

The study involved two steps: initially, a systematic review to identify moral distress scales conducted in animal care worker. By scale, we understand any standardized questionnaire, some of which could be a PROM (Patient-Reported Outcome Measures). PROM is standardized, validated questionnaires/rating scales that are completed by patients to capture their perceptions of their health, exposure, and quality of life [22]. The second step focused on assessing the psychometric validity of these PROMs, as per COSMIN guidelines [23]. The results were reported according to the PRISMA (Preferred Reporting Items for Systematic Reviews and Meta-Analysis) [24] and COSMIN guidelines [23].

Step 1. Systematic review of the moral distress measurements used in animal care workers

For peer review only - http://bmjopen.bmj.com/site/about/guidelines.xhtml

The study protocol was conducted according to protocol registered in Prospective Register of Systematic Reviews (PROSPERO) under the registration number (CRD42023422259). We preregistered this review to specify the research plan and design, to ensure discoverability of the study progress, and to report the results systematically. By preregistering this review, we wanted to overcome our confirmation and hindsight biases to the topic [25].

Eligibility criteria

In this review we included the studies meeting the following criteria: 1) original (primary) study conducted in animal care workers, 2) describing either the development of a moral distress measure, or validation of a moral distress measure in its original or modified version, 3) published in English, German, French, Turkish, or Russian languages. We excluded: 1) reviews, editorials, commentaries, and conference papers, 2) studies using or assessing other outcomes than moral distress, and 3) grey literature.

Data sources and search terms

We used three databases: PubMed, EMBASE, and PsycINFO to search for eligible studies published between January 1984 and April 2023. An experienced librarian developed the research strategy that consisted of free-text words to define three search strings, the terms focusing on the population of interest (i.e., animal care workers), terms related to moral distress, and terms related to PROMs. The latter was facilitated using the application of a sensitive filter developed by Terwee et al. [20]. Reference lists were manually checked to identify additional studies, during the second screening of full texts of included studies.

Study screening and selection

The librarian imported all items retrieved from each of the three databases into the bibliography software EndNote 20 and removed the duplicates. The items were then imported into the Rayyan application [26]

For peer review only

For peer review only - http://bmjopen.bmj.com/site/about/guidelines.xhtml

for screening. The screening of eligible studies was conducted by two independent reviewers [Y.B, N.G] in two steps. The first screening was conducted based on the title and abstract according to the criteria described above. The second screening was based on full text reading where the same inclusion criteria were applied. All eligible or unclassifiable items were included in the second screening, conducted based on the full-text reading. In both steps, reviewers discussed the discrepancies, and when necessary, they consulted a third reviewer [I.G.C]. As part of the quality control, 25% of the items in both steps of screening were cross-checked by a fourth independent reviewer with expertise in veterinary research [S.H.].

Step 2. Systematic review of psychometric validity of the identified moral distress scales

In the second step of the systematic review, measurement properties of PROMs are assessed using the COSMIN checklist for methodological quality of studies on measurement properties [21]. Assessing the methodological quality of PROMs is essential to determine the adequacy of the use of a PROM in a given target population. The COSMIN checklist outlines the necessary design standards and statistical approaches and allows selection of best PROM by comparing design requirements, sound methodological steps taken, and reporting of studies that develop and validate PROMs [21]. The checklist is made up from taxonomy of main measurement prosperities of validity, reliability, and responsiveness; which includes 12 sub-measurement properties [21]. The foremost step in evaluating the adequacy of PROMs according to the COSMIN methodology is content validity assessment since it directly examines whether the instrument reflects the specific domain of interest from the perspective of the target population [20]. Content validity property confirms the ability of the PROMs to be adequate reflection of the construct they measure. This review was conducted according to the COSMIN guidelines for systematic reviews of PROMs [20, 21, 23] and the practical guidelines on the identification of the most valid PROM [27]. These guidelines suggest an ordering to evaluate measurement properties of PROMs, starting with content validity. Since PROMs rely on reported outcomes of a given target population in their scale items, a degree of confidence needs to be cleared on whether the items of the PROM are relevant, comprehensive, and

For peer review only - http://bmjopen.bmj.com/site/about/guidelines.xhtml

comprehensible with respect to the construct of interest and target population [19]. Later, pooled scores of evaluation of all measurement properties are adjusted based on their quality of evidence using modified GRADE approach for risk of bias [19, 23].

Before evaluating any psychometric properties, the user manual [19] recommends searching relevant PROMs in COSMIN database of systematic reviews of moral distress scales in the target population to overcome redundancy. We searched in the COSMIN database for the systematic reviews on moral distress scales in our target population [20].

Data extraction and synthesis

We considered the following information relevant for extraction: study reference, name of the moral distress scale used, psychometric properties assessed, and methods and results of their assessments. Whenever necessary the reviewers contacted the study authors to complete this information. The collected information was then summarized in a narrative synthesis.

Patient and Public Involvement Statement

Patients or the public were not involved in the design, or conduct, or reporting, or dissemination plans of our research.

Results

Included studies

Step 1

The literature search resulted in 560 items. After duplicates removal, 455 records were retained for the first screening based on titles and abstracts. For the second screening based on the full-text screening, we selected 16 articles, from which only one met the inclusion criteria (Figure 1). In the other articles, either

For peer review only - http://bmjopen.bmj.com/site/about/guidelines.xhtml

the moral distress was not measured (n=13), or it was an ineligible type of publication: such as a dissertation (n=1), or the article did not satisfy the inclusion criteria (n=1), only using the moral distress prevalence as one of the covariates.

The included study was published in 2023 by Kogan et al. [15] and aimed to assess moral distress in veterinarians, differentiating between the veterinarians and the animal owners. Kogan et al. found that gender and age were predictors of moral distress. Indeed, the level of moral distress was higher in young women than in older men. Moreover, the authors found that a high level of moral distress was associated with a low level of professional fulfillment and with a high level of exhaustion, burnout, and interpersonal disengagement [15].

Step 2

In the second step, we evaluated the included studies based on the COSMIN methodology checklist [21]. Following the COSMIN approach, no systematic review on animal health care taker was found in the COSMIN database, but an existing systematic review for human health care professionals assessed 32 moral distress scales in their original, revised, and translated versions [18]. Our current systematic review and the previous systematic review by Gianetta et al. [18 have certain similarities in their research questions, objectives, and study design. In both of our research questions/ objectives, we tried to identify measures of moral distress and evaluate them according to COSMIN methodology. Both of the reviews were conducted in a two-steps systematic review procedure. In both of the reviews, adaptations of the original studies were identified and evaluated separately.

Nevertheless, despite Gianetta et al.'s claim of adhering to COSMIN guidelines in their review, the evaluation of the content validity of existing moral distress PROMs was not thoroughly executed. Therefore, three reviewers [Y.B, N.G, I.G.C] took it upon themselves to assess the content validity of the PROMs identified in the initial step. This fortuitous discovery within Gianetta et al.'s review, however, led us to the

For peer review only

For peer review only - http://bmjopen.bmj.com/site/about/guidelines.xhtml

original PROM (MMD-HP) [28], from which our chosen scale, MMD-AP [15], was derived. By assessing the original PROM (MMD-HP) along with the adapted version (MMD-AP), we were able to better capture the content of originally selected items and assess their relevance and comprehensiveness with respect to the theoretical foundations of moral distress construct features.

In the COSMIN framework, all revised versions of a PROM are considered as new PROMs and should be assessed independently. As PROMs measure constructs that can only be reported by the patients themselves, no gold standards exist for these measures [19]. In cases when PROM is adapted to be used in another target population than the original target population for which it was developed, a new evaluation is necessary to appraise the relevancy of items in the original PROM [21]. As an example, a PROM that is originally developed for moral distress in human health care professionals may not be relevant for veterinary professionals. Human health care and veterinary medicine may experience different moral dilemmas and constraints, and deal with different stakeholders. While an item may reflect on moral distress related to "convenience euthanasia" due to animal owner's request, an original PROM from human health care would not include this potentially relevant item.

Therefore, in the second step of this study, we considered both PROMs: MMD-HP and MMD-AP for associates and animal owners. We chose the MMD-AP version of associates over the practice owners because the associates' version is the same as the owners' version but contains three more items. These items are related to superiors' pressure and their lack of support. Thus, we think this version is more representative of the animal workers' job experience and covers all items in the both MMD-AP versions. Consequently, we concluded that since no qualitative studies conducted for item selection of these adapted scales in two different populations, there is no justification for a separate assessment.

Construct definition and PROM description

For peer review only - http://bmjopen.bmj.com/site/about/guidelines.xhtml

The two identified PROMs share the same construct of Moral distress, initially defined by Jameton [13]. Epstein et al. [28] defined it later as follows: *"The pressure to act unethically is the defining concept of the phenomenon and it separates those situations that are emotionally distressing or otherwise morally troubling from those that threaten moral integrity"*. Epstein et al. [28] considered five important factors as causes of moral distress that inspired the MMD-HP structure: complicity in wrongdoing, lack of voice, professional values, the repetitive nature of moral distress, and the three roots of causes. These three roots were ordered at three different levels: patients or their families, for example when the family asks for a treatment that does more harm to the patient than good. The colleagues or team can be a cause of moral distress, for example in the case of poor communication between team members. Finally, the system can cause moral distress when there is a lack of human, material, or financial resources. Moral distress can be a combination of stressors arising at these three levels. For instance, if the worker faces pressure situations in which he/she cannot report an error committed by a colleague frequently, his/her opinion cannot be considered and his/her professional ethic could be violated [28].

Measure of Moral Distress for Health Professionals (MMD-HP)

The first identified PROM, MMD-HP was developed in 2019, by revising the Moral Distress Scale Revised (MDS-R) [29]. Epstein et al. revised MDS-R to make it applicable to all health workers. MDS-R itself originally stems from the Moral Distress Scale (MDS) [30]. Epstein et al. underwent the construction of a new PROM to overlook the drawbacks of the original and previous revised versions, namely to simplify the use and better integrate the causal levels of moral distress, more specifically, the team and system levels. Epstein et al. reviewed the literature over the past five years to identify new roots of causes of moral distress. The new elements led to the deletion of three items, the modification of three other items, the consolidation of two items, and the addition of 11 new items. The new items integrated the team and system levels, which according to Epstein et al., were a weakness of MDS-R, mostly focused on the patient level. The MMD-HP contains 27 items and the three levels of roots to separate the items into four factors. Some items appear in more than one factor. The first factor corresponds to the primarily system level, while the second factor

For peer review only - http://bmjopen.bmj.com/site/about/guidelines.xhtml

corresponds to the clinical level at the patient level. Factors 3 and 4 both represent the team level. However, factor 3 contains items, which imply a threat to personal integrity from a member of his team, whereas factor 4 concerns the team's communication with patients and families.

The MMD-HP intends a double scaling, one for the frequency of the described situations and one for the degree of distress each situation results in. Both are scored on a 5-point Likert scale, ranging from 0 (never) to 4 (very frequently) for the frequency and from 0 (none) to 4 (very distressing) for the degree of distress. The product of the frequency and the distress scores corresponds to an overall score of moral distress, ranging from 0 to 432. The higher the score, the greater the level of moral distress. However, the authors did not mention any cut-off values [28].

Measure of Moral Distress for Animal Professionals (MMD-AP)

The second PROM, MMD-AP resulted from an additional revision of the MMD-HP. Kogan et al. (14) used the MMD-HP [28] to assess moral distress in veterinarians. The associates' version, for employed veterinarians, includes 24 items, and the owners' version has 21 items. The authors explained that they removed items, which did not fit the veterinary context. The authors considered the five components at three levels, described by Epstein et al. [28], which were included in the MMD-AP [15]; these components are complicity in wrongdoing, lack of voice, wrongdoing associated with professional (not personal) values, repeated experiences, and three levels of root causes (at patient, unit, system level) [28]. For example, for the root causes system level, one item reads: "*Experience lack of administrative action or support for a problem that is compromising patient care"* [15]. For the patient level root causes, an item reads: *"Euthanasia based on client's unwillingness to treat"*. For the team level, there is an item: *"Feel pressured by superiors to order or carry out orders for what I consider to be unnecessary or inappropriate tests and treatments"* [15]. For each item, the response scale is based on the frequency and the distress experienced. Response options and calculation of the moral distress overall score are the same as the MMD-HP.

Quality of psychometric validity of the selected PROMs

Quality of the PROM development

For peer review only - http://bmjopen.bmj.com/site/about/guidelines.xhtml

The COSMIN guidelines for evaluating the quality of the study on the PROM development consist of two parts. The first part deals with the standards used to assess the quality of the searches carried out to find the most suitable items for the new PROM. It includes general design requirements and concept elicitation. The concept of elicitation is important for relevance and comprehensiveness of the PROM. The second part looks at the standards used to assess the quality of the cognitive interview study or pilot studies conducted. The aim is to rate the comprehensiveness and comprehensibility of the PROM [20].

For both PROMs, the construct and its origin, the target population, and the context of use were well-defined and rated as very good. Both PROMs have been developed in English and tested in a sample representing the target population, thus the general design requirements were also rated as very good. We appraised the concept elicitation as doubtful for MMD-AP and adequate for MMD-HP. For these two PROMs, we could not rate them as very good, because the qualitative data collection was not or not enough described, even after contacting the authors for further information on the analyses they carried out. The total PROM design was thus rated as adequate for MMD-HP and doubtful for MMD-AP. The MMD-AP authors conducted a very good cognitive interview asking the target population (i.e., veterinarians) for guidance and feedback, thus we evaluated the general design requirements as very good. For MMD-HP, the rating was adequate because the cognitive interview was performed with experts and clinicians but not with all health professionals. However, Kogan et al. did not ask the participants about the comprehensibility of the items included in the PROM. Comprehensibility is the standard that ensures that PROM is comprehensible to the target population. For a very good rating, different PROM components need to be evaluated separately: instruction, item, response option, and recall period [20]. Since the authors did not mention whether they have evaluated these components for MMD-AP, we rated the quality of the comprehensibility study as doubtful. Finally, the total quality of the MMD-AP development study was rated as doubtful. For MMD-HP, the comprehensibility was also assessed as doubtful. Although its authors

For peer review only - http://bmjopen.bmj.com/site/about/guidelines.xhtml

carried out a study to evaluate the face validity, they did not mention whether they used an interview guide and did verbatim transcriptions.

Table 1 summarizes the results of the PROM development assessment, with the items considered and their consensual rating by two independent reviewers.

Quality of the PROM content validity

The criteria to assess content validity are relevance, comprehensiveness, and comprehensibility. The relevance means, for example, ensuring that PROM items are adapted to the target population and to the context in which they are used. The comprehensiveness evaluates the completeness of the PROM and the comprehensibility concerns the understanding of the PROM by the target population. Table 2 summarizes the results of these three criteria for PROMs content validity assessment.

Quality of evidence for moral distress scales are assessed using the modified GRADE approach for risk of bias [19, 23]. This approach allows reviewers to adjust their evaluations of pooled results of PROM measurement property evaluations. It assumes that pooled results of individual measurement properties have an overestimation of the quality of evidence; thus, they have to be downgraded, given suspicion over trustworthiness of the published results. It consists of five factors to determine the quality of the evidence: risk of bias, inconsistency, indirectness, imprecision, and publication bias. Within these factors, three of these factors are applicable to content validity evaluation: risk of bias, inconsistency, and indirectness [19, 20]. Risk of bias depends on the number of publications available in the literature on a given PROM on its development to numerous studies assessing its measurement properties, and their ratings by reviewers based on their adequacy. Indirectness factor is related to whether qualitative methods are conducted to a representative sample of the intended target population. The inconsistency factor depends on the variability of rating given to each item by reviewers in COSMIN content validity methodology checklist. If there is no explanation found to explain the variability of ratings, the rating is downgraded to “inconsistent” [19].

For peer review only - http://bmjopen.bmj.com/site/about/guidelines.xhtml

Table 3 summarizes the results of the PROMs overall rating of content validity, with the adjusted quality of evidence.

For the MMD-AP content validity assessment, the development study was evaluated as inconsistent. The lack of information on qualitative data collection for the development of this PROM led to an insufficient assessment of the relevance. For comprehensiveness, the key concepts were missing, and we rated it as insufficient. The comprehensibility was also rated as insufficient because the authors did not report whether they tested or not the understanding of the PROM. Both reviewers rated the development study of this PROM as sufficient. The reviewers estimated that the PROM itself contained the necessary information. Consequently, the overall rating of the MMD-AP was inconsistent. There was no content validity study available for this PROM [15]. Therefore, the lack of studies on content validity and the doubtful PROM development study has resulted in a low quality of evidence.

The content validity of MMD-HP was rated as sufficient. Indeed, the relevance, comprehensiveness, and comprehensibility were rated as sufficient, and the two reviewers also evaluated these properties as sufficient. Overall, the content validity rating was also sufficient. Since we did not find any content validity study for MMD-HP, the quality of evidence grading started as moderate. Then, we downgraded it to low because the PROM's development study has been assessed as doubtful.

Other psychometric properties of the selected PROMs

According to the COSMIN framework, in case the PROM does not meet the criteria for a sufficient quality of content validity, the assessment of other psychometric proprieties is useless, the content validity being a paramount requirement for recommending a PROM for use in research or clinical practice. The content validity was inconsistent for MDD-AP and sufficient for MDD-HP, therefore, we did not conduct any formal assessment of the other properties of these PROMs. Nevertheless, Gianetta et al. made a systematic review of studies assessing additional psychometric properties of MDD-HP [18]. The internal consistency

For peer review only

For peer review only - http://bmjopen.bmj.com/site/about/guidelines.xhtml

of MDD-HP was graded as sufficient, with a Cronbach alpha of 0.93 on average [18]. The structural validity was appraised as sufficient. It was assessed by an exploratory factor analysis, where the four identified factors accounted for 54.3% of the variance [18]. Nonetheless, no confirmatory factor analysis was performed, contrary to the COSMIN guidelines for assessing the quality of structural validity [18]. Gianetta et al. evaluated the cross-cultural validity and hypothesis testing as sufficient. The reliability was appraised as sufficient with the inter-rater agreement of 88% [18]. Gianetta et al. didn't report any analysis concerning measurement error, responsiveness, and construct validity of MMD-HP. Finally, they judged the criterion validity as lacking.

Discussion

Main findings

This study focused on identifying valid Patient-Reported Outcome Measures (PROMs) to assess moral distress in animal care workers. A two-step systematic review revealed the scarcity of standardized measures in this field. Only one specific measure for veterinarians, the Measure of Moral Distress for Animal Professionals (MMD-AP), was identified and evaluated for content validity following COSMIN guidelines [15, 19]. This finding was unexpected, particularly given the substantial number of studies on moral issues among animal care workers (i.e., 455 records in our databases). Striking also was the fact that very few studies used standardized questionnaires or PROM for the moral distress measurements, i.e., the most important eligibility criteria in our systematic review. This points out a need to create awareness in this research field regarding the importance of a valid and reliable outcome measurement, using high-quality, validated methods.

In our evaluation based on COSMIN guidelines, we found the quality of MMD-AP development to be doubtful, with inconsistent content validity and low-quality evidence [20]. "Doubtful" ratings in our findings do not suggest an ordinal measure of PROM quality in its development; rather, they suggest lack

For peer review only - http://bmjopen.bmj.com/site/about/guidelines.xhtml

of qualitative methods applied to develop and to evaluate PROMs, and poor reporting of application of these methods.

The findings highlight several critical issues. Firstly, moral distress in animal care workers has not received adequate attention compared to other outcomes like anxiety, compassion fatigue, or burnout. Secondly, many studies claiming to measure moral distress did not follow a valid or standardized methodology. Thirdly, there seems to be a disregard for proper PROM development and psychometric validation methods in current research in this area. This gap in methodological rigor hinders understanding the nature, determinants, and prevention of moral distress among animal care workers. However, this issue is not unique to animal care but is also prevalent in other fields, as demonstrated by the review of Gianetta et al [18]. Their review, included in the COSMIN database, did not assess the content validity of the reviewed PROMs as required by COSMIN guidelines, reflecting a broader trend of methodological shortcomings in current research practices.

The only PROM available for measuring moral distress in animal care workers is the MMD-AP [15]. Due to its current inconsistent content validity and low-quality evidence, further studies are necessary for its validation and improvement. Before its reassessment, the MMD-AP is not recommended for use in research or clinical practice, as it fails to meet COSMIN criteria of relevance, comprehensiveness, and comprehensibility.

Improving the quality of MMD-AP's content validity assessment requires qualitative studies involving the target population, including animal shelter workers, lab workers, and veterinary nurses. This could result in changes or the introduction of new items, leading to an updated version of the MMD-AP that would necessitate validation studies.

Limitations and Strengths

Certain limitations must be acknowledged, inherently dependent to the design and execution of this review.

For peer review only - http://bmjopen.bmj.com/site/about/guidelines.xhtml

Firstly, COSMIN guidelines provide only a loose directory in cases where reporting of qualitative studies is poor. It gives only general rules for content validity studies, defining what must be reported. It does not provide guidance on how much detail is required to meet the criteria of the COSMIN methodology for assessing content validity [31]. Indeed, COSMIN provides guidelines for an indirect assessment of PROM quality by methodological quality of development and subsequent validation studies of PROMs. Thus, reporting of qualitative studies is one of the main indicators of methodological quality of PROMs [21]. Although COSMIN guidelines precise which methodological steps must be reported, there is no guidance on how much details they should be. Due to this shortcoming, it is often that qualitative studies are insufficiently reported, and rated as “doubtful” [31].

Secondly, development studies of PROMs in this review suffers from novelty of COSMIN guidelines. While they started to appear in late 2010s, majority of original development of moral distress scales date to early 2000s [29, 30], before the publication of these guidelines. This may explain the lack of qualitative studies and patient involvement. Yet, while we did see those qualitative methods being introduced for human healthcare professionals, these methods are lacking for veterinary medicine practitioners.

Lastly, moral distress has been coined 40 years ago in the nursing discipline and since then further definitions have been developed, possibly leading to a heterogeneous and complex understanding of moral distress. However, since COSMIN evaluation relies on a degree of subjectivity by reviewers to rate the standards of criteria [20], not specific enough definitions and operationalizations may endanger evaluation of PROMs without additional qualitative studies [32]. For example, we encountered both moral stress and moral distress in our first review. Moreover, while we observed that there are many studies focusing on conceptual aspects of moral issues among animal care workers, very few studies used standardized questionnaires or PROM for the moral distress measurements. The use of standardized questionnaires was a criterion for the eligibility in this review. Hence, in the end, we could only identify and evaluate one specific scale for animal care workers. A recent study by Buchbinder et al. (2023) focused on differential assessment of moral stress and moral distress [33]. They discuss that moral stress is linked to systemic

For peer review only

For peer review only - http://bmjopen.bmj.com/site/about/guidelines.xhtml

overburdening and does not necessarily involve feelings of powerlessness, contrary to moral distress. Since moral distress pays more attention to clinical encounters to specific stressors, rather than systemic origins of stressors in healthcare; it is relatively easy to develop items that reflect those clinical encounters to develop a standardized questionnaire. At the same time, current moral distress scales may suggest that moral distress is an individual pathology, rather than a complex syndrome, due to the lack of attention given to systematic constraints.

To deal with this subjective nature of rating, we relied on the transparent and systemic methodology of COSMIN [20]. The study followed PRISMA guidelines and pre-registered with PROSPERO. This ensures a thorough, transparent, and traceable methodological approach. The adherence to COSMIN guidelines for assessing the content validity of PROMs ensures a rigorous evaluation of the measurement properties of outcome measures. Our evaluation conducted on every version of the scale; and informed by a clearly articulated clinical or research question(s), the methodological quality of PROM design, the development process, findings of the content validity study, and content of the scale itself. By synthesizing robust assessment of COSMIN framework with transparent methodological approach, this study primes the design and standardization of valid MD assessments.

Interpretations

An accurate assessment of a health problem in terms of incidence or prevalence requests a validated standard for measuring this problem in clinical, occupational, or research settings. It could ideally be a validated diagnostic test, or a consensually approved set of diagnostic criteria, such as those used for quantifying the burden of major depressive disorder or general anxiety disorder in the global burden of diseases [34]. Unless such tests or criteria have been established, PROMs can help estimate the amount of the problem, as it was recently shown in occupational burnout research [35]. Yet, the prerequisite for this is an appropriate choice of the most valid PROM [36, 37]. Compared to occupational burnout, moral distress is a relatively recent construct in occupational psychology, therefore, the research on moral distress could

For peer review only - http://bmjopen.bmj.com/site/about/guidelines.xhtml

benefit from and capitalize on the methodological development in subjective outcome measurement in the other fields.

Conclusion

This is the first systematic review focused on moral distress measurement in animal care workers. It showed that moral distress is rarely measured using standardized and evidence-based methods. These findings underline the need to develop and thoroughly validate such methods in the context of animal care. We think it is also necessary for the well-being of the animals, as we believe that moral distress has an impact on the animals cared for by the animal caretakers. workers. More generally, methodological improvement and training in psychometry are recommended when developing and validating scales of moral distress and other subjective outcomes.

Figure Legends

Figure 1. Flow-chart of study selection

Declarations

- **Word count:** 5679

- **Ethics approval and consent to participate**

  Not applicable.

- **Consent for publication**

  Not applicable.

- **Availability of data and materials**

For peer review only - http://bmjopen.bmj.com/site/about/guidelines.xhtml

Data are available online as supplemental material of the present article.

- **Competing interests**

None declared.

- **Funding**

This project has received funding from Swiss National Science Foundation (SNF) for the NRP79 project 407940_206396 “Linking animal and human welfare – refining rodent euthanasia”.

- **Authors' contributions**

Conceptualization, I.G.C.; methodology, N.G., Y.B., and I.G.C.; software, N.G. and Y.B.; validation, N.G., Y.B., I.G.C. and S.H; formal analysis, N.G., and Y.B.; investigation, N.G., Y.B., and I.G.C.; resources, N.G., Y.B., and I.G.C.; data curation, N.G., Y.B., and I.G.C.; writing—original draft preparation, N.B., Y.B., and I.G.C; writing—review and editing, N.G., Y.B., I.G.C. and S.H.; supervision, I.G.C. and S.H ; project administration, I.G.C. and S.H; funding acquisition, I.G.C and S.H. All authors have read and agreed to the published version of the manuscript.

- **Acknowledgements**

The authors thank the Unisanté documentarist, Thomas Brauchly, for his precious help in establishing the literature search queries.

For peer review only

References

1. World Health Organization. WHO guidelines on mental health at work. Geneva: World Health Organization; 2022.
2. Wang H, Abbas KM, Abbasifard M, Abbasi-Kangevari M, Abbastabar H, Abd-Allah F, et al. Global age-sex-specific fertility, mortality, healthy life expectancy (HALE), and population estimates in 204 countries and territories, 1950–2019: a comprehensive demographic analysis for the Global Burden of Disease Study 2019. Lancet. 2020;396(10258):1160-203. doi: 10.1016/S0140-6736(20)30977-6
3. Crane MF, Phillips JK, Karin E. Trait perfectionism strengthens the negative effects of moral stressors occurring in veterinary practice. Aust Vet J. 2015;93(10):354-60. doi: 10.1111/avj.12366.
4. Shoman Y, Rousson V, Bianchi R, Guseva Canu I. Holistic Assessment of Factors Associated with Exhaustion, the Main Symptom of Burnout: A Meta-Analysis of Longitudinal Studies. Int J Environ Res Public Health. 2022;19(20). doi: 10.3390/ijerph192013037.
5. Al-Gobari M, Shoman Y, Blanc S, Canu IG. Point prevalence of burnout in Switzerland: a systematic review and meta-analysis. Swiss Med Wkly. 2022; 152:w30229. doi: 10.4414/smw.2022.w30229.
6. Burdorf A. How to improve intervention research on the psychosocial work environment? Scand J Work Environ Health. 2023;49(5):311-3. doi: 10.5271/sjweh.4107.
7. Zimmermann C, Strohmaier S, Niederkrotenthaler T, Thau K, Schernhammer E. Suicide mortality among physicians, dentists, veterinarians, and pharmacists as well as other high-skilled occupations in Austria from 1986 through 2020. Psychiatry Res. 2023;323:115170. doi:10.1016/j.psychres.2023.115170
8. Moses L, Malowney MJ, Wesley Boyd J. Ethical conflict and moral distress in veterinary practice: A survey of North American veterinarians. J Vet Intern Med. 2018 ;32(6):2115-22. doi: 10.1111/jvim.15315
9. Arbe Montoya AI, Hazel S, Matthew SM, McArthur ML. Moral distress in veterinarians. Vet Rec. 2019;185(20):631. doi: 10.1136/vr.105289.
10. LaFollette MR, Riley MC, Cloutier S, Brady CM, O'Haire ME, Gaskill BN. Laboratory Animal Welfare Meets Human Welfare: A Cross-Sectional Study of Professional Quality of Life, Including Compassion Fatigue, Among Laboratory Animal Personnel. Qual Life Res. 2018; 27(5):1147-57. doi.org/10.3389/fvets.2020.00114
11. Scotney RL, McLaughlin D, Keates HL. A systematic review of the effects of euthanasia and occupational stress in personnel working with animals in animal shelters, veterinary clinics, and biomedical research facilities. J Am Vet Med Assoc. 2015;247(10):1121-30. doi: 10.2460/javma.247.10.1121
12. Grimm H, Biller-Andorno N, Buch T, et al. Advancing the 3Rs: innovation, implementation, ethics and society. Front Vet Sci. 2023;10:1185706. doi:10.3389/fvets.2023.1185706
13. Jameton A. Dilemmas of moral distress: moral responsibility and nursing practice. AWHONNS Clin Issues Perinat Womens Health Nurs. 1993;4(4):542-51.
14. Jameton A, Jackson EM. Nuclear war and nursing ethics. What is the nurse's responsibility? Möbius: J Contin Educ Prof Health Sci. 1984;4(1):75-88. DOI: 10.1002/chp.4760040110

For peer review only - http://bmjopen.bmj.com/site/about/guidelines.xhtml

15. Kogan LR, Rishniw M. Veterinarians and moral distress. J Am Vet Med Assoc. 2023;261(5):1-7. doi: 10.2460/javma.22.12.0598.
16. Reeve C, Rogelberg S, Spitzmueller C, Digiacomo N. The Caring-Killing Paradox: Euthanasia-Related Strain Among Animal-Shelter Workers. J Appl Soc Psychol. 2006;35:119-43. DOI: 10.1111/j.1559-1816.2005.tb02096.x
17. Andrukonis A, Hall NJ, Protopopova A. The Impact of Caring and Killing on Physiological and Psychometric Measures of Stress in Animal Shelter Employees: A Pilot Study. Int J Environ Res Public Health. 2020;17(24). DOI: 10.3390/ijerph17249196.
18. Giannetta N, Villa G, Pennestrì F, Sala R, Mordacci R, Manara DF. Instruments to assess moral distress among healthcare workers: A systematic review of measurement properties. Int J Nurs Stud. 2020; 111:103767. doi: 10.1016/j.ijnurstu.2020.103767.
19. Mokkink LB, de Vet HCW, Prinsen CAC, Patrick DL, Alonso J, Bouter LM, et al. COSMIN Risk of Bias checklist for systematic reviews of Patient-Reported Outcome Measures. Qual Life Res. 2018;27(5):1171-9. DOI: 10.1007/s11136-017-1765-4
20. Terwee CB, Prinsen CAC, Chiarotto A, Westerman MJ, Patrick DL, Alonso J, et al. COSMIN methodology for evaluating the content validity of patient-reported outcome measures: a Delphi study. Qual Life Res. 2018;27(5):1159-70. DOI: 10.1007/s11136 -018-1829-0
21. Mokkink, L.B., Terwee, C.B., Knol, D.L. et al. The COSMIN checklist for evaluating the methodological quality of studies on measurement properties: A clarification of its content. BMC Med Res Methodol 10, 22 (2010). https://doi.org/10.1186/1471-2288-10-22
22. Kingsley C, Patel S. Patient-reported outcome measures and patient-reported experience measures. BJA Educ. 2017;17(4):137-44. DOI: 10.1093/bjaed/mkw060
23. Prinsen CAC, Mokkink LB, Bouter LM, Alonso J, Patrick DL, de Vet HCW, et al. COSMIN guideline for systematic reviews of patient-reported outcome measures. Qual Life Res. 2018;27(5):1147-57. doi: 10.1007/s11136-018-1798-3.
24. Moher D, Liberati A, Tetzlaff J, Altman DG. Preferred reporting items for systematic reviews and meta-analyses: the PRISMA statement. PLoS Med. 2009;6(7):e1000097. doi:10.1371/journal.pmed.1000097.
25. Nosek BA, Ebersole CR, DeHaven AC, Mellor DT. The preregistration revolution. Proc Natl Acad Sci U S A. 2018;115(11):2600-2606. doi:10.1073/pnas.1708274114
26. Ouzzani M, Hammady H, Fedorowicz Z, Elmagarmid A. Rayyan-a web and mobile app for systematic reviews. Syst Rev. 2016;5(1):210. doi.org/10.1186/s13643-016-0384-4
27. Shoman Y, Majery N, Otelea M, Lambreghts C, Guseva Canu I. How to identify the most suitable questionnaires and rating scales for your clinical practice or research? Int J Clin Pract. 2021;75(12): e14895. doi: 10.1111/ijcp.14895
28. Epstein EG, Whitehead PB, Prompahakul C, Thacker LR, Hamric AB. Enhancing Understanding of Moral Distress: The Measure of Moral Distress for Health Care Professionals. AJOB Empir Bioeth. 2019;10(2):113-24. doi: 10.1080/23294515.2019.1586008.
29. Hamric AB. Empirical research on moral distress: issues, challenges, and opportunities. HEC Forum. 2012;24(1):39-49. doi: 10.1007/s10730-012-9177-x.
30. Corley MC, Elswick RK, Gorman M, Clor T. Development and evaluation of a moral distress scale. J Adv Nurs. 2001;33(2):250-6. doi: 10.1046/j.1365-2648.2001.01658.x.

For peer review only - http://bmjopen.bmj.com/site/about/guidelines.xhtml

31. Rothmund, M., Meryk, A., Rumpold, G. et al. A critical evaluation of the content validity of patient-reported outcome measures assessing health-related quality of life in children with cancer: a systematic review. J Patient Rep Outcomes 7, 2 (2023). DOI: https://doi.org/10.1186/s41687-023-00540-8
32. van Andel EM, Koopmann BDM, Crouwel F, Noomen CG, de Boer NKH, van Asseldonk DP, Mokkink LBM. Systematic Review of Development and Content Validity of Patient reported Outcome Measures in Inflammatory Bowel Disease: Do We Measure What We Measure?. J Crohn's Colitis. 2020;14(9):1299–1315. https://doi.org/10.1093/ecco-jcc/jjaa057
33. Buchbinder M, Browne A, Berlinger N, Jenkins T, Buchbinder L. Moral Stress and Moral Distress: Confronting Challenges in Healthcare Systems under Pressure. Am J Bioeth. 2023. doi:10.1080/15265161.2023.2224270
34. Santomauro DF, Mantilla Herrera AM, Shadid J, Zheng P, Ashbaugh C, Pigott DM, et al. Global prevalence and burden of depressive and anxiety disorders in 204 countries and territories in 2020 due to the COVID-19 pandemic. Lancet. 2021;398(10312):1700-12. DOI: https://doi.org/10.1016/S0140-6736(21)02143-7
35. Guseva Canu I, Marca SC, Dell'Oro F, Balázs Á, Bergamaschi E, Besse C, et al. Harmonized definition of occupational burnout: A systematic review, semantic analysis, and Delphi consensus in 29 countries. Scand J Work Environ Health. 2021;(2):95-107. doi: 10.5271/sjweh.3935.
36. Guseva Canu I, Mesot O, Györkös C, Mediouni Z, Mehlum IS, Bugge MD. Burnout syndrome in Europe: towards a harmonized approach in occupational health practice and research. Ind Health. 2019;57(6):745-52. doi: 10.2486/indhealth.2018-0159.
37. Shoman Y, Marca S, Bianchi R, Godderis L, Molen HF, Guseva Canu I. Psychometric properties of burnout measures: A systematic review. Epidemiol Psychiatr Sci. 2021;30:e8. doi: 10.1017/S2045796020001134.

For peer review only - http://bmjopen.bmj.com/site/about/guidelines.xhtml

Table 1. Quality of the PROM development

**PROM**	**MMD-HP**	**MMD-AP**
First author, year	Esptein et al., 2019	Kogan et al., 2023
Language in which the PROM was developed	English	English
**PROM design**		
Clear construct	V	V
Clear origin of construct	V	V
Clear target population for which the PROM was developed	V	V
Clear context of use	V	V
PROM developed in sample representing the target population	V	V
Concept elicitation	A	D
Total PROM design	***A***	***D***
Cognitive interview study performed in sample representing the target population	A	V
Comprehensibility	D	D
Total Cognitive interview study	***D***	***D***
**Total PROM development**	**D**	**D**

Note*: V: very good, A: adequate, D: doubtful*

For peer review only

Table 2. Systematic results for the MMD-HP and MMD-AP PROMs for the criteria content validity

PROM	Development study	Rating of reviewers	Overall rating + / - / ?	Quality of evidence
**MMD-HP**				
Relevance	+	±	+	**Low**
Comprehensiveness	+	+	+	**Low**
Comprehensibility	+	+	+	**Low**
**Content validity rating**	+	+	+	**Low**
**MMD-AP**				
Relevance	±	+	±	**Low**
Comprehensiveness	-	+	±	**Low**
Comprehensibility	-	+	±	**Low**
**Content validity rating**	±	+	±	**Low**

Note: +*: sufficient, -: insufficient,* ±*: inconsistent*

For peer review only

Table 3. Systematic results for the overall rating of MMD-HP and MMD-AP PROMs and their quality of evidence

**PROM**	**MMD-HP**		**MMD-AP**	
**COSMIN psychometric properties for content validity**	**Overall rating + / - / ?**	**Quality of evidence High, moderate, low, very low**	**Overall rating + / - / ?**	**Quality of evidence High, moderate, low, very low**
Relevance	+	**Low**	±	**Low**
Comprehensiveness	+	**Low**	-	**Low**
Comprehensibility	+	**Low**	-	**Low**

Note: +*: sufficient, -: insufficient,* ±: *inconsistent*

For peer review only

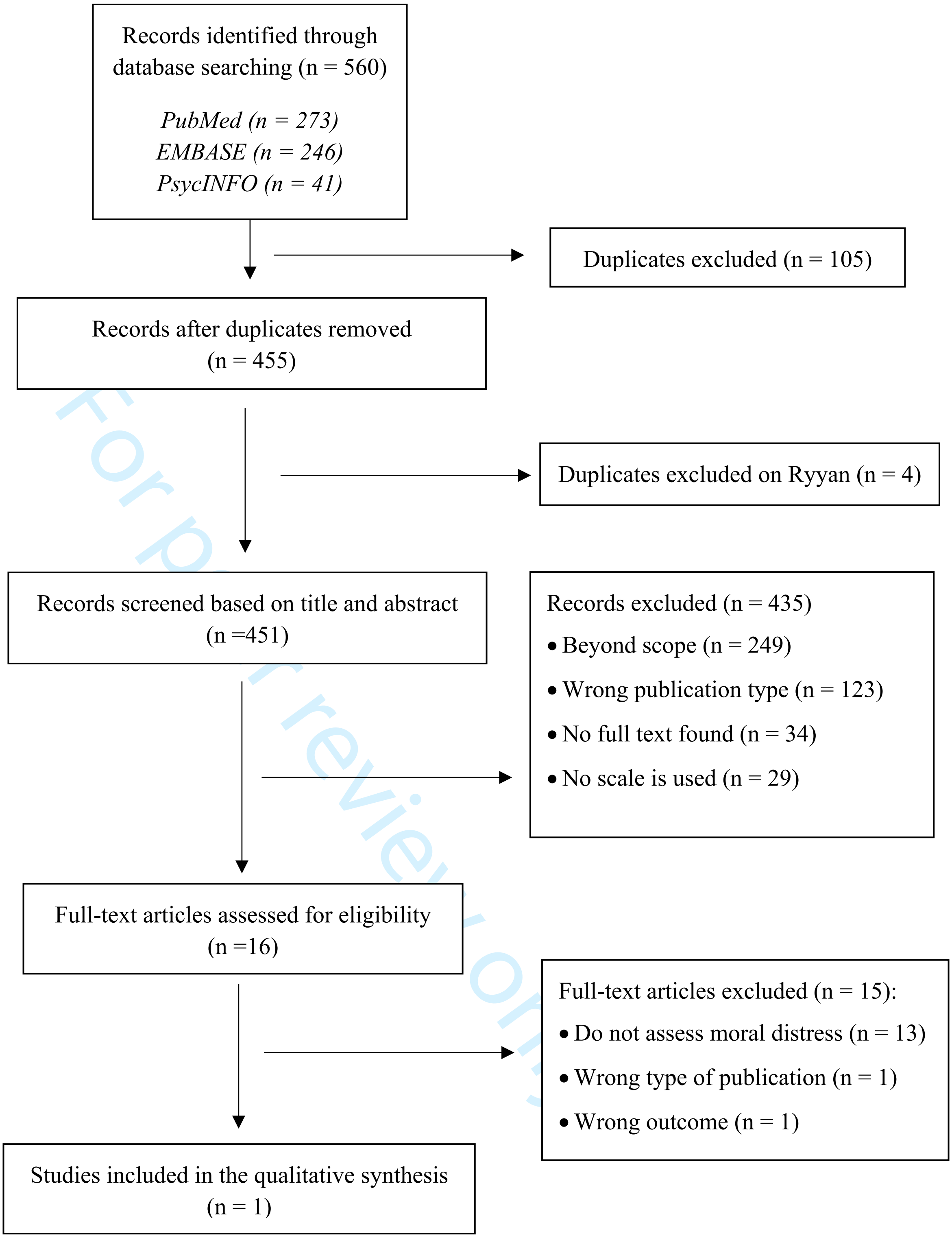

Figure 1: Flow-chart

For peer review only - http://bmjopen.bmj.com/site/about/guidelines.xhtml

PRISMA 2020 Checklist

Section and Topic	Item #	Checklist item	Location where item is reported
**TITLE**			
Title	1	Identify the report as a systematic review.	1
**ABSTRACT**			
Abstract	2	See the PRISMA 2020 for Abstracts checklist.	21
**INTRODUCTION**			
Rationale	3	Describe the rationale for the review in the context of existing knowledge.	68
Objectives	4	Provide an explicit statement of the objective(s) or question(s) the review addresses.	118
**METHODS**			
Eligibility criteria	5	Specify the inclusion and exclusion criteria for the review and how studies were grouped for the syntheses.	141
Information sources	6	Specify all databases, registers, websites, organisations, reference lists and other sources searched or consulted to identify studies. Specify the date when each source was last searched or consulted.	147
Search strategy	7	Present the full search strategies for all databases, registers and websites, including any filters and limits used.	Supplemental materials
Selection process	8	Specify the methods used to decide whether a study met the inclusion criteria of the review, including how many reviewers screened each record and each report retrieved, whether they worked independently, and if applicable, details of automation tools used in the process.	155
Data collection process	9	Specify the methods used to collect data from reports, including how many reviewers collected data from each report, whether they worked independently, any processes for obtaining or confirming data from study investigators, and if applicable, details of automation tools used in the process.	158
Data items	10a	List and define all outcomes for which data were sought. Specify whether all results that were compatible with each outcome domain in each study were sought (e.g. for all measures, time points, analyses), and if not, the methods used to decide which results to collect.	189
	10b	List and define all other variables for which data were sought (e.g. participant and intervention characteristics, funding sources). Describe any assumptions made about any missing or unclear information.	Not applicable
Study risk of bias assessment	11	Specify the methods used to assess risk of bias in the included studies, including details of the tool(s) used, how many reviewers assessed each study and whether they worked independently, and if applicable, details of automation tools used in the process.	184
Effect measures	12	Specify for each outcome the effect measure(s) (e.g. risk ratio, mean difference) used in the synthesis or presentation of results.	Tables 1,2 and 3
Synthesis methods	13a	Describe the processes used to decide which studies were eligible for each synthesis (e.g. tabulating the study intervention characteristics and comparing against the planned groups for each synthesis (item #5)).	192
	13b	Describe any methods required to prepare the data for presentation or synthesis, such as handling of missing summary statistics, or data conversions.	Not applicable
	13c	Describe any methods used to tabulate or visually display results of individual studies and syntheses.	Not applicable
	13d	Describe any methods used to synthesize results and provide a rationale for the choice(s). If meta-analysis was performed, describe the model(s), method(s) to identify the presence and extent of statistical heterogeneity, and software package(s) used.	Not applicable
	13e	Describe any methods used to explore possible causes of heterogeneity among study results (e.g. subgroup analysis, meta-regression).	Not applicable
	13f	Describe any sensitivity analyses conducted to assess robustness of the synthesized results.	Not

For peer review only - http://bmjopen.bmj.com/site/about/guidelines.xhtml

PRISMA 2020 Checklist

Section and Topic	Item #	Checklist item	Location where item is reported
			applicable
Reporting bias assessment	14	Describe any methods used to assess risk of bias due to missing results in a synthesis (arising from reporting biases).	Not applicable
Certainty assessment	15	Describe any methods used to assess certainty (or confidence) in the body of evidence for an outcome.	Not applicable
**RESULTS**			
Study selection	16a	Describe the results of the search and selection process, from the number of records identified in the search to the number of studies included in the review, ideally using a flow diagram.	200
	16b	Cite studies that might appear to meet the inclusion criteria, but which were excluded, and explain why they were excluded.	204
Study characteristics	17	Cite each included study and present its characteristics.	206
Risk of bias in studies	18	Present assessments of risk of bias for each included study.	346/ Table 3
Results of individual studies	19	For all outcomes, present, for each study: (a) summary statistics for each group (where appropriate) and (b) an effect estimate and its precision (e.g. confidence/credible interval), ideally using structured tables or plots.	Tables 1,2 and 3
Results of syntheses	20a	For each synthesis, briefly summarise the characteristics and risk of bias among contributing studies.	331 /Table 2
	20b	Present results of all statistical syntheses conducted. If meta-analysis was done, present for each the summary estimate and its precision (e.g. confidence/credible interval) and measures of statistical heterogeneity. If comparing groups, describe the direction of the effect.	Not applicable
	20c	Present results of all investigations of possible causes of heterogeneity among study results.	Not applicable
	20d	Present results of all sensitivity analyses conducted to assess the robustness of the synthesized results.	Not applicable
Reporting biases	21	Present assessments of risk of bias due to missing results (arising from reporting biases) for each synthesis assessed.	Not applicable
Certainty of evidence	22	Present assessments of certainty (or confidence) in the body of evidence for each outcome assessed.	Not applicable
**DISCUSSION**			
Discussion	23a	Provide a general interpretation of the results in the context of other evidence.	456
	23b	Discuss any limitations of the evidence included in the review.	414
	23c	Discuss any limitations of the review processes used.	Not applicable
	23d	Discuss implications of the results for practice, policy, and future research.	470
**OTHER INFORMATION**			
Registration and protocol	24a	Provide registration information for the review, including register name and registration number, or state that the review was not registered.	51
	24b	Indicate where the review protocol can be accessed, or state that a protocol was not prepared.	51
	24c	Describe and explain any amendments to information provided at registration or in the protocol.	Not

For peer review only - http://bmjopen.bmj.com/site/about/guidelines.xhtml

PRISMA 2020 Checklist

Section and Topic	Item #	Checklist item	Location where item is reported
			applicable
Support	25	Describe sources of financial or non-financial support for the review, and the role of the funders or sponsors in the review.	52
Competing interests	26	Declare any competing interests of review authors.	486
Availability of data, code and other materials	27	Report which of the following are publicly available and where they can be found: template data collection forms; data extracted from included studies; data used for all analyses; analytic code; any other materials used in the review.	Supplemental materials

From: Page MJ, McKenzie JE, Bossuyt PM, Boutron I, Hoffmann TC, Mulrow CD, et al. The PRISMA 2020 statement: an updated guideline for reporting systematic reviews. BMJ 2021;372:n71. doi: 10.1136/bmj.n71

For more information, visit: http://www.prisma-statement.org/

For peer review only

BMJ Open

Moral distress measurement in animal care workers: a systematic review

Journal:	*BMJ Open*
Manuscript ID	bmjopen-2023-082235.R2
Article Type:	Original research
Date Submitted by the Author:	01-Apr-2024
Complete List of Authors:	Baysal, Yigit; University of Zurich Vetsuisse Faculty, Section of Epidemiology<br>Goy, Nastassja; University of Lausanne, Department of occupational and environmental health<br>Hartnack, Sonja; University of Zurich Vetsuisse Faculty, Section of Epidemiology<br>Canu, Irina ; Unisanté, Department of occupational and environmental health
**Primary Subject Heading**:	Qualitative research
Secondary Subject Heading:	Public health, Occupational and environmental medicine, Nursing, Mental health
Keywords:	Behavior, Psychological Stress < Stress, Psychological, Psychometrics, Occupational Stress, ETHICS (see Medical Ethics)

SCHOLARONE™
Manuscripts

I, the Submitting Author has the right to grant and does grant on behalf of all authors of the Work (as defined in the below author licence), an exclusive licence and/or a non-exclusive licence for contributions from authors who are: i) UK Crown employees; ii) where BMJ has agreed a CC-BY licence shall apply, and/or iii) in accordance with the terms applicable for US Federal Government officers or employees acting as part of their official duties; on a worldwide, perpetual, irrevocable, royalty-free basis to BMJ Publishing Group Ltd ("BMJ") its licensees and where the relevant Journal is co-owned by BMJ to the co-owners of the Journal, to publish the Work in this journal and any other BMJ products and to exploit all rights, as set out in our licence.

The Submitting Author accepts and understands that any supply made under these terms is made by BMJ to the Submitting Author unless you are acting as an employee on behalf of your employer or a postgraduate student of an affiliated institution which is paying any applicable article publishing charge ("APC") for Open Access articles. Where the Submitting Author wishes to make the Work available on an Open Access basis (and intends to pay the relevant APC), the terms of reuse of such Open Access shall be governed by a Creative Commons licence – details of these licences and which Creative Commons licence will apply to this Work are set out in our licence referred to above.

Other than as permitted in any relevant BMJ Author's Self Archiving Policies, I confirm this Work has not been accepted for publication elsewhere, is not being considered for publication elsewhere and does not duplicate material already published. I confirm all authors consent to publication of this Work and authorise the granting of this licence.

For peer review only

Moral distress measurement in animal care workers: a systematic review

Yigit Baysal[1*, **], Nastassja Goy[2*], Sonja Hartnack[1], Irina Guseva Canu[2]

[1] Section of Epidemiology, Vetsuisse Faculty, University of Zurich, Zurich, Switzerland

[2]Department of occupational and environmental health, Unisanté, University of Lausanne, Lausanne, Switzerland

* Equally contributing authors

** Corresponding author. Email: yigit.baysal@uzh.ch; Address: Section of Epidemiology, Vetsuisse Faculty, University of Zurich, Winterthurerstrasse 270, CH-8057, Zürich, Switzerland

For peer review only - http://bmjopen.bmj.com/site/about/guidelines.xhtml

Abstract

Objectives: The mental health of veterinary and other animal health professionals is significantly impacted by the psychological stressors they encounter, such as euthanasia, witnessing animal suffering, and moral distress. Moral distress, initially identified in nursing, arises when individuals are aware of the right action but are hindered by institutional constraints. We aimed to review existing research on moral distress scales among animal care workers, by focusing on the identification and psychometric validity of its measurement.

Design: Two-step systematic review. First, we identified all moral distress scales used in animal care research, in the eligible original studies. Second, we evaluated their psychometric validity, emphasizing content validity, which is a critical aspect of patient-reported outcome measures (PROMs). This evaluation adhered to the Consensus-based Standards for the Selection of Health Measurement Instruments (COSMIN). The results were reported according to the PRISMA.

Data sources: PubMed, EMBASE, and PsycINFO to search for eligible studies published between January 1984 and April 2023.

Eligibility criteria for selecting studies: We included original (primary) studies that 1) were conducted in animal care workers; describing 2) either the development of a moral distress scale, or validation of a moral distress scale in its original or modified version, to assess at least one of the psychometric properties mentioned in COSMIN guidelines.

Data extraction and synthesis Two independent reviewers used standardized methods to search, screen and code included studies. We considered the following information relevant for extraction: study reference, name and reference of the moral distress scale used, psychometric properties assessed, and methods and results of their assessments. The collected information was then summarized in a narrative synthesis.

For peer review only

For peer review only - http://bmjopen.bmj.com/site/about/guidelines.xhtml

Results: The review identified only one PROM specifically adapted for veterinary contexts: the Measure of Moral Distress for Animal Professionals (MMD-AP), derived from the Measure of Moral Distress for Healthcare Professionals (MMD-HP). Both MMD-HP and MMD-AP were evaluated for the quality of development and content validity. The development quality of both measures was deemed doubtful. According to COSMIN, MMD-HP's content validity was rated as sufficient, whereas MMD-AP's was inconsistent. However, the evidence quality for both PROMs was rated low.

Conclusion: This is the first systematic review focused on moral distress measurement in animal care workers. It shows that moral distress is rarely measured using standardized and evidence-based methods and that such methods should be developed and validated in the context of animal care.

PROSPERO registration number: CRD42023422259

Funding: This project has received funding from Swiss National Science Foundation (SNF) for the NRP79 project 407940_206396 “Linking animal and human welfare – refining rodent euthanasia”.

Keywords: Ethics; content validity; psychometric properties; patient-reported outcome measures (PROMs); occupational health.

Strengths and Limitations

- The adherence to COSMIN guidelines for assessing the content validity of PROMs ensures rigorous evaluation of the scales of moral distress (MD) and their psychometric validity.
- The strength of the review's conclusions is inherently dependent on the quality and robustness of the original studies included.
- Moral distress was coined in 1984 in the nursing discipline and since then further definitions have been developed, possibly leading to a heterogeneous and complex understanding of moral distress.

For peer review only - http://bmjopen.bmj.com/site/about/guidelines.xhtml

Background

Animal care workers' mental health and work stressors

The World Health Organization (WHO) defines mental health as a state of well-being essential for coping with daily life and engaging in meaningful work, beyond just the absence of mental disorders [1]. The Global Burden of Disease (GBD) study highlights a bidirectional relationship between work and mental health [2]. Participating in meaningful work serves as a protective factor for mental health, while mental well-being influences occupational functioning and quality of professional life [1]. However, work-related stressors, particularly those with moral challenges, can negatively impact mental health [3]. Occupational mental health issues like burnout, compassion fatigue, and moral distress (MD) have been studied in healthcare workers but less so in other professions facing similar stressors, such as animal care workers [4,5,6]. Animal care workers, including lab technicians and veterinarians, are at risk of mental ill-health and suicide, particularly due to unique stressors like euthanasia [4,5,7,8,9]. Compared to human healthcare professionals, research on moral distress among animal care workers is limited.

Both human and animal health care professionals, directly or indirectly being responsible for the care of their patients, form emotional bonds with them. However, at the same time, they may perform procedures that may cause pain and distress to their patients or find themselves unable to alleviate their pain and distress. Occupational mental health of animal care professionals has recently been emphasized, i.e., La Follette et al. suggested that animal care professionals are significantly experiencing emotional dissonance and moral stress [10]. A key difference between human healthcare and animal care professionals regarding workplace stress lies in the outcomes within clinical and experimental settings. The endpoint in animal experimentation is typically the death of the animal. In harm-benefit analysis it is crucially important that attempts are made to minimize harm and suffering in animal experimental setting. This ethical framework is often in conflict with the predisposition of attachment, love and empathy based on human-animal bonds. Animal care workers often find themselves constantly making and breaking these emotional bonds [10].

For peer review only - http://bmjopen.bmj.com/site/about/guidelines.xhtml

Combined with the role conflict experienced due to balancing competing interests of animal welfare and human interests, moral dilemma experienced at the workplace can transform into moral distress [8]. Social support networks can buffer the effects of moral dilemma and role conflict by providing emotional support and alternative perspectives that help the individual navigate ethical dilemmas and role demands. Yet, a systematic review of workplace stress in animal care workers found reports of qualitative studies where stigma associated with animal related work tasks (e.g., euthanasia, use of animals in research) can hinder the access of social support networks [11]. Considering the complexity of moral distress in animal care work, we emphasize the need for exploration of this psychological syndrome targeted to animal care workers.

Moreover, experiencing moral distress and its negative consequences potentially impacts animal welfare, including the implementation of the 3R principle (replacement, reduction, refinement) in the animal experimental setting with animal care takers being responsible for fostering these [12]. An association between poorer quality of life in laboratory animal personnel and limited control over euthanasia procedures was found [10].

Concept of moral distress and its attributes

Moral distress was first described in nursing as "when one knows the right thing to do, but institutional constraints make it nearly impossible to pursue the right course of action" [13, 14]. It involves complicity in wrongdoing, lack of voice, and conflict with professional values, often exacerbated in hierarchical work environments [13, 14, 15]. In veterinary medicine, moral distress arises from practices like euthanasia and witnessing animal suffering, with personal experiences and attitudes towards animals influencing its development. Veterinarians also report high levels of compassion fatigue, anxiety, burnout, depression, and suicidal thoughts [7,8,9]. Moral distress affects both professional and personal quality of life and can exacerbate PTSD, burnout, and compassion fatigue [9, 16, 17].

For peer review only - http://bmjopen.bmj.com/site/about/guidelines.xhtml

Despite several systematic reviews on moral distress in medical workers, none have specifically addressed animal care workers [18]. Various tools, mostly self-administered questionnaires, or Patient-Reported Outcome Measures (PROMs) [19] have been developed to measure moral distress. Front of such diversity, a question of their relevance and validity in the context of animal care practice arises.

Study objectives

In this study, we aimed to systematically review the available literature to answer two complementary research questions: 1-Which scales or PROMs are used in animal health care workers to assess moral distress? and 2-What is the psychometric quality of these PROMs? The quality of a PROM is defined by its validity, reliability, and responsiveness, with the content validity being the most important psychometric property [19]. According to the COnsensus-based Standards for the selection of health Measurement INstruments (COSMIN) [19, 20, 21], the content validity is "the degree to which the content of a scale is an adequate reflection of the construct to be measured" [20]. Thus, we focused on the content validity, while assessing the quality of the identified moral distress measures.

Methods

The study involved two steps: initially, a systematic review to identify moral distress scales conducted in animal care worker. By scale, we understand any standardized questionnaire, some of which could be a PROM (Patient-Reported Outcome Measures). PROM is a standardized, validated questionnaires/rating scales that are completed by patients to capture their perceptions of their health, exposure, and quality of life [22]. The second step focused on assessing the psychometric validity of these PROMs, as per COSMIN guidelines [23]. The results were reported according to the PRISMA (Preferred Reporting Items for Systematic Reviews and Meta-Analysis) [24] and COSMIN guidelines [23].

Step 1. Systematic review of the moral distress measurements used in animal care workers

For peer review only - http://bmjopen.bmj.com/site/about/guidelines.xhtml

The study protocol was conducted according to protocol registered in Prospective Register of Systematic Reviews (PROSPERO) under the registration number (CRD42023422259). We preregistered this review to specify the research plan and design, to ensure discoverability of the study progress, and to report the results systematically. By preregistering this review, we wanted to overcome our confirmation and hindsight biases to the topic [25].

Eligibility criteria

In this review we included the studies meeting the following criteria: 1) original (primary) study conducted in animal care workers, 2) describing either the development of a moral distress measure, or validation of a moral distress measure in its original or modified version, 3) published in English, German, French, Turkish, or Russian languages. We excluded: 1) reviews, editorials, commentaries, and conference papers, 2) studies using or assessing other outcomes than moral distress, and 3) grey literature.

Data sources and search terms

We used three databases: PubMed, EMBASE, and PsycINFO to search for eligible studies published between January 1984 and April 2023. In the initial phase of planning our study, we considered reviewing literature over an arbitrary but long period of 50 years. However, upon discovering the term "moral distress" was first coined by Jameton in 1984, we adjusted our review period to start from that year onwards. An experienced librarian developed the research strategy that consisted of free-text words to define three search strings, the terms focusing on the population of interest (i.e., animal care workers), terms related to moral distress, and terms related to PROMs. The latter was facilitated using the application of a sensitive filter developed by Terwee et al. [20]. Reference lists were manually checked to identify additional studies, during the second screening of full texts of included studies.

Study screening and selection

For peer review only - http://bmjopen.bmj.com/site/about/guidelines.xhtml

The librarian imported all items retrieved from each of the three databases into the bibliography software EndNote 20 and removed the duplicates. The items were then imported into the Rayyan application [26] for screening. The screening of eligible studies was conducted by two independent reviewers [Y.B, N.G] in two steps. The first screening was conducted based on the title and abstract according to the criteria described above. The second screening was based on full text reading where the same inclusion criteria were applied. All eligible or unclassifiable items were included in the second screening, conducted based on the full-text reading. In both steps, reviewers discussed the discrepancies, and when necessary, they consulted a third reviewer [I.G.C], as suggested by COSMIN guidelines [20]. As part of the quality control, 25% of the items in both steps of screening were cross-checked by a fourth independent reviewer with expertise in veterinary research [S.H.].

Step 2. Systematic review of psychometric validity of the identified moral distress scales

In the second step of the systematic review, measurement properties of PROMs are assessed using the COSMIN checklist for methodological quality of studies on measurement properties [21]. Assessing the methodological quality of PROMs is essential to determine the adequacy of the use of a PROM in a given target population. The COSMIN checklist outlines the necessary design standards and statistical approaches and allows selection of best PROM by comparing design requirements, sound methodological steps taken, and reporting of studies that develop and validate PROMs [21]. The checklist is made up from the taxonomy of main measurement properties of validity, reliability, and responsiveness which includes 12 sub-measurement properties [21]. The foremost step in evaluating the adequacy of PROMs according to the COSMIN methodology is content validity assessment since it directly examines whether the instrument reflects the specific domain of interest from the perspective of the target population [20]. Content validity property confirms the ability of the PROMs to be an adequate reflection of the construct they measure. This review was conducted according to the COSMIN guidelines for systematic reviews of PROMs [20, 21, 23] and the practical guidelines on the identification of the most valid PROM [27]. These

For peer review only

For peer review only - http://bmjopen.bmj.com/site/about/guidelines.xhtml

guidelines suggest an ordering to evaluate measurement properties of PROMs, starting with content validity. As PROMs base their scale items on reported outcomes of a specific target population, a certain level of confidence in the relevance, completeness and comprehensibility of the PROM items in relation to the construct of interest and the target population needs to be clarified [19]. Later, pooled scores of evaluations of all measurement properties are adjusted based on their quality of evidence using the modified GRADE approach for risk of bias [19, 23].

Before evaluating any psychometric properties, the user manual [19] recommends searching relevant PROMs in the COSMIN database of systematic reviews of moral distress scales in the target population to overcome redundancy. We searched in the COSMIN database for the systematic reviews on moral distress scales in our target population [20].

Data extraction and synthesis

We considered the following information relevant for extraction: study reference, name of the moral distress scale used, psychometric properties assessed, and methods and results of their assessments. Whenever necessary the reviewers contacted the study authors to complete this information. The collected information was then summarized in a narrative synthesis.

Patient and Public Involvement Statement

Patients or the public were not involved in the design, or conduct, or reporting, or dissemination plans of our research.

Results

Included studies

Step 1

For peer review only - http://bmjopen.bmj.com/site/about/guidelines.xhtml

The literature search resulted in 560 items. After duplicate removal, 455 records were retained for the first screening based on titles and abstracts. For the second screening based on the full-text screening, we selected 16 articles, from which only one met the inclusion criteria (Figure 1). In the other articles, either the moral distress was not measured (n=13), or it was an ineligible type of publication: such as a dissertation (n=1), or the article did not satisfy the inclusion criteria (n=1), only using the moral distress prevalence as one of the covariates.

The included study was published in 2023 by Kogan et al. [15] and aimed to assess moral distress in veterinarians, differentiating between the owners of veterinary practices and the employed associates. Kogan et al. found that gender and age were predictors of moral distress. Indeed, the level of moral distress was higher in young women than in older men. Moreover, the authors found that a high level of moral distress was associated with a low level of professional fulfillment and with a high level of exhaustion, burnout, and interpersonal disengagement [15].

Step 2

In the second step, we evaluated the included studies based on the COSMIN methodology checklist [21]. Following the COSMIN approach, no systematic review on animal health care taker was found in the COSMIN database, but an existing systematic review for human health care professionals assessed 32 moral distress scales in their original, revised, and translated versions [18]. Our current systematic review and the previous systematic review by Gianetta et al. [18] have certain similarities in their research questions, objectives, and study design. In both of our research questions/ objectives, we tried to identify measures of moral distress and evaluate them according to COSMIN methodology. Both of the reviews were conducted in a two-steps systematic review procedure. In both of the reviews, adaptations of the original studies were identified and evaluated separately.

For peer review only - http://bmjopen.bmj.com/site/about/guidelines.xhtml

Nevertheless, despite Gianetta et al.'s claim of adhering to COSMIN guidelines in their review, the evaluation of the content validity of existing moral distress PROMs was not thoroughly executed. Therefore, three reviewers [Y.B, N.G, I.G.C] took it upon themselves to assess the content validity of the PROMs identified in the initial step. This fortuitous discovery within Gianetta et al.'s review, however, led us to the original PROM (MMD-HP) [28], from which our chosen scale, MMD-AP [15], was derived. By assessing the original PROM (MMD-HP) along with the adapted version (MMD-AP), we were able to better capture the content of originally selected items and assess their relevance and comprehensiveness with respect to the theoretical foundations of moral distress construct features.

In the COSMIN framework, all revised versions of a PROM are considered as new PROMs and should be assessed independently. As PROMs measure constructs that can only be reported by the patients themselves, no gold standards exist for these measures [19]. In cases when PROM is adapted to be used in another target population than the original target population for which it was developed, a new evaluation is necessary to appraise the relevancy of items in the original PROM [21]. As an example, a PROM that is originally developed for moral distress in human health care professionals may not be relevant for veterinary professionals. Human health care and veterinary medicine may experience different moral dilemmas and constraints, and deal with different stakeholders. While an item may reflect moral distress related to “convenience euthanasia” due to animal owner’s request, an original PROM from human health care would not include this potentially relevant item.

Therefore, in the second step of this study, we considered both PROMs: MMD-HP and MMD-AP for owners of veterinary practices and the employed associates. We chose the MMD-AP version of associates over the practice owners because the associates’ version is the same as the owners’ version but contains three more items. These items are related to superiors’ pressure and their lack of support. Thus, we think this version is more representative of the animal workers’ job experience and covers all items in the both MMD-AP versions. Consequently, we concluded that since no qualitative studies were conducted for item

For peer review only - http://bmjopen.bmj.com/site/about/guidelines.xhtml

selection of these adapted scales in two different populations, there is no justification for a separate assessment.

Construct definition and PROM description

The two identified PROMs share the same construct of Moral distress, initially defined by Jameton [13]. Epstein et al. [28] defined it later as follows: *"The pressure to act unethically is the defining concept of the phenomenon and it separates those situations that are emotionally distressing or otherwise morally troubling from those that threaten moral integrity"*. Epstein et al. [28] considered five important factors as causes of moral distress that inspired the MMD-HP structure: complicity in wrongdoing, lack of voice, professional values, the repetitive nature of moral distress, and the three roots of causes. These three roots were ordered at three different levels: patients or their families, for example when the family asks for a treatment that does more harm to the patient than good. The colleagues or the team can be a cause of moral distress, for example in the case of poor communication between team members. Finally, the system can cause moral distress when there is a lack of human, material, or financial resources. Moral distress can be a combination of stressors arising at these three levels. For instance, if the worker faces pressure situations in which he/she cannot report an error committed by a colleague frequently, his/her opinion cannot be considered and his/her professional ethic could be violated [28].

Measure of Moral Distress for Health Professionals (MMD-HP)

The first identified PROM, MMD-HP was developed in 2019, by revising the Moral Distress Scale Revised (MDS-R) [29]. Epstein et al. revised MDS-R to make it applicable to all health workers. MDS-R itself originally stems from the Moral Distress Scale (MDS) [30]. Epstein et al. underwent the construction of a new PROM to amend the drawbacks of the original and previous revised versions, namely to simplify the use and to better integrate the causal levels of moral distress, more specifically, the team and system levels. Epstein et al. reviewed the literature over the past five years to identify new roots of causes of moral distress. The new elements led to the deletion of three items, the modification of three other items, the consolidation

For peer review only - http://bmjopen.bmj.com/site/about/guidelines.xhtml

of two items, and the addition of 11 new items. The new items integrated the team and system levels, which according to Epstein et al., were a weakness of MDS-R, mostly focused on the patient level. The MMD-HP contains 27 items and the three levels of roots to separate the items into four factors. Some items appear in more than one factor. The first factor corresponds to the primarily system level, while the second factor corresponds to the clinical level at the patient level. Factors 3 and 4 both represent the team level. However, factor 3 contains items, which imply a threat to personal integrity from a member of his team, whereas factor 4 concerns the team's communication with patients and families.

The MMD-HP intends a double scaling, one for the frequency of the described situations and one for the degree of distress each situation results in. Both are scored on a 5-point Likert scale, ranging from 0 (never) to 4 (very frequently) for the frequency and from 0 (none) to 4 (very distressing) for the degree of distress. The product of the frequency and the distress scores corresponds to an overall score of moral distress, ranging from 0 to 432. The higher the score, the greater the level of moral distress. However, the authors did not mention any cut-off values [28].

Measure of Moral Distress for Animal Professionals (MMD-AP)

The second PROM, MMD-AP resulted from an additional revision of the MMD-HP. Kogan et al. (14) used the MMD-HP [28] to assess moral distress in veterinarians. The associates' version, for employed veterinarians, includes 24 items, and the owners' version has 21 items. The authors explained that they removed items, which did not fit the veterinary context. The authors considered the five components at three levels, described by Epstein et al. [28], which were included in the MMD-AP [15]; these components are complicity in wrongdoing, lack of voice, wrongdoing associated with professional (not personal) values, repeated experiences, and three levels of root causes (at patient, unit, system level) [28]. For example, for the root causes system level, one item reads: "*Experience lack of administrative action or support for a problem that is compromising patient care*" [15]. For the patient level root causes, an item reads: *"Euthanasia based on client's unwillingness to treat"*. For the team level, there is an item: *"Feel pressured by superiors to order or carry out orders for what I consider to be unnecessary or inappropriate tests and*

For peer review only - http://bmjopen.bmj.com/site/about/guidelines.xhtml

treatments" [15]. For each item, the response scale is based on the frequency and the distress experienced. Response options and calculation of the moral distress overall score are the same as the MMD-HP.

Quality of psychometric validity of the selected PROMs

Quality of the PROM development

The COSMIN guidelines for evaluating the quality of the study on the PROM development consist of two parts. The first part deals with the standards used to assess the quality of the searches carried out to find the most suitable items for the new PROM. It includes general design requirements and concept elicitation. The concept of elicitation is important for relevance and comprehensiveness of the PROM. The second part looks at the standards used to assess the quality of the cognitive interview study or pilot studies conducted. The aim is to rate the comprehensiveness and comprehensibility of the PROM [20].

For both PROMs, the construct and its origin, the target population, and the context of use were well-defined and rated as very good. Both PROMs have been developed in English and tested in a sample representing the target population, thus the general design requirements were also rated as very good. We appraised the concept elicitation as doubtful for MMD-AP and adequate for MMD-HP. For these two PROMs, we could not rate them as very good, because the qualitative data collection was not or not enough described, even after contacting the authors for further information on the analyses they carried out. The total PROM design was thus rated as adequate for MMD-HP and doubtful for MMD-AP. The MMD-AP authors conducted a very good cognitive interview asking the target population (i.e., veterinarians) for guidance and feedback, thus we evaluated the general design requirements as very good. For MMD-HP, the rating was adequate because the cognitive interview was performed with experts and clinicians but not with all health professionals. However, Kogan et al. did not ask the participants about the comprehensibility of the items included in the PROM. Comprehensibility is the standard that ensures that PROM is

For peer review only - http://bmjopen.bmj.com/site/about/guidelines.xhtml

comprehensible to the target population. For a very good rating, different PROM components need to be evaluated separately: instruction, item, response option, and recall period [20]. Since the authors did not mention whether they have evaluated these components for MMD-AP, we rated the quality of the comprehensibility study as doubtful. Finally, the total quality of the MMD-AP development study was rated as doubtful. For MMD-HP, the comprehensibility was also assessed as doubtful. Although its authors carried out a study to evaluate the face validity, they did not mention whether they used an interview guide and did verbatim transcriptions.

Table 1 summarizes the results of the PROM development assessment, with the items considered and their consensual rating by two independent reviewers.

Quality of the PROM content validity

The criteria to assess content validity are relevance, comprehensiveness, and comprehensibility. The relevance means, for example, ensuring that PROM items are adapted to the target population and to the context in which they are used. The comprehensiveness evaluates the completeness of the PROM and the comprehensibility concerns the understanding of the PROM by the target population. Table 2 summarizes the results of these three criteria for PROMs content validity assessment.

Quality of evidence for moral distress scales are assessed using the modified GRADE approach for risk of bias [19, 23]. This approach allows reviewers to adjust their evaluations of pooled results of PROM measurement property evaluations. It assumes that pooled results of individual measurement properties have an overestimation of the quality of evidence; thus, they have to be downgraded, given suspicion over trustworthiness of the published results. It consists of five factors to determine the quality of the evidence: risk of bias, inconsistency, indirectness, imprecision, and publication bias. Within these factors, three of these factors are applicable to content validity evaluation: risk of bias, inconsistency, and indirectness [19, 20]. The risk of bias depends on the number of publications available in the literature on a given PROM, from its development to numerous studies evaluating its measurement properties, and the evaluators' judgements based on their appropriateness. For example, the indirectness factor is related to whether

For peer review only - http://bmjopen.bmj.com/site/about/guidelines.xhtml

347 qualitative methods are carried out on a representative sample of the intended target population. The
348 inconsistency factor depends on the variability of ratings given to each item by reviewers based on the
349 COSMIN content validity methodology checklist. If there is no explanation found to explain the variability
350 of ratings, the rating is downgraded to "inconsistent" [19].

351 Table 3 summarizes the results of the PROMs overall rating of content validity, with the adjusted quality
352 of evidence.

353

354 For the MMD-AP content validity assessment, the development study was evaluated as inconsistent. The
355 lack of information on qualitative data collection for the development of this PROM led to an insufficient
356 assessment of the relevance. For comprehensiveness, the key concepts were missing, and we rated it as
357 insufficient. The comprehensibility was also rated as insufficient because the authors did not report whether
358 they tested or not the understanding of the PROM. Both reviewers rated the development study of this
359 PROM as sufficient. The reviewers estimated that the PROM itself contained the necessary information.
360 Consequently, the overall rating of the MMD-AP was inconsistent. There was no content validity study
361 available for this PROM [15]. Therefore, the lack of studies on content validity and the doubtful PROM
362 development study has resulted in a low quality of evidence.

363 The content validity of MMD-HP was rated as sufficient. Indeed, the relevance, comprehensiveness, and
364 comprehensibility were rated as sufficient, and the two reviewers also evaluated these properties as
365 sufficient. Overall, the content validity rating was also sufficient. Since we did not find any content validity
366 study for MMD-HP, the quality of evidence grading started as moderate. Then, we downgraded it to low
367 because the PROM's development study has been assessed as doubtful.

368 *Other psychometric properties of the selected PROMs*

For peer review only - http://bmjopen.bmj.com/site/about/guidelines.xhtml

According to the COSMIN framework, in case the PROM does not meet the criteria for a sufficient quality of content validity, the assessment of other psychometric proprieties is useless, the content validity being a paramount requirement for recommending a PROM for use in research or clinical practice. The content validity was inconsistent for MDD-AP and sufficient for MDD-HP, therefore, we did not conduct any formal assessment of the other properties of these PROMs. Nevertheless, Gianetta et al. made a systematic review of studies assessing additional psychometric properties of MDD-HP [18]. The internal consistency of MDD-HP was graded as sufficient, with a Cronbach alpha of 0.93 on average [18]. The structural validity was appraised as sufficient. It was assessed by an exploratory factor analysis, where the four identified factors accounted for 54.3% of the variance [18]. Nonetheless, no confirmatory factor analysis was performed, contrary to the COSMIN guidelines for assessing the quality of structural validity [18]. Gianetta et al. evaluated the cross-cultural validity and hypothesis testing as sufficient. The reliability was appraised as sufficient with the inter-rater agreement of 88% [18]. Gianetta et al. didn't report any analysis concerning measurement error, responsiveness, and construct validity of MMD-HP. Finally, they judged the criterion validity as lacking.

Discussion

Main findings

This study focused on identifying valid Patient-Reported Outcome Measures (PROMs) to assess moral distress in animal care workers. A two-step systematic review revealed the scarcity of standardized measures in this field. Only one specific measure for veterinarians, the Measure of Moral Distress for Animal Professionals (MMD-AP), was identified and evaluated for content validity following COSMIN guidelines [15, 19]. This finding was unexpected, particularly given the substantial number of studies on moral issues among animal care workers (i.e., 455 records in our databases). Striking also was the fact that very few studies used standardized questionnaires or PROM for the moral distress measurements, i.e., the most important eligibility criteria in our systematic review. This points out a need to create awareness in

For peer review only - http://bmjopen.bmj.com/site/about/guidelines.xhtml

393 this research field regarding the importance of a valid and reliable outcome measurement, using high-
394 quality, validated methods.

395 In our evaluation based on COSMIN guidelines, we found the quality of MMD-AP development to be
396 doubtful, with inconsistent content validity and low-quality evidence [20]. "Doubtful" ratings in our
397 findings do not suggest an ordinal measure of PROM quality in its development; rather, they suggest lack
398 of qualitative methods applied to develop and to evaluate PROMs, and poor reporting of application of
399 these methods.

400 The findings highlight several critical issues. Firstly, moral distress in animal care workers has not received
401 adequate attention compared to other outcomes like anxiety, compassion fatigue, or burnout. Secondly,
402 many studies claiming to measure moral distress did not follow a valid or standardized methodology.
403 Thirdly, there seems to be a disregard for proper PROM development and psychometric validation methods
404 in current research in this area. This gap in methodological rigor hinders understanding the nature,
405 determinants, and prevention of moral distress among animal care workers. However, this issue is not
406 unique to animal care but is also prevalent in other fields, as demonstrated by the review of Gianetta et al
407 [18]. Their review, included in the COSMIN database, did not assess the content validity of the reviewed
408 PROMs as required by COSMIN guidelines, reflecting a broader trend of methodological shortcomings in
409 current research practices.

410 The only PROM available for measuring moral distress in animal care workers is the MMD-AP [15]. Due
411 to its current inconsistent content validity and low-quality evidence, further studies are necessary for its
412 validation and improvement. Before its reassessment, the MMD-AP is not recommended for use in research
413 or clinical practice, as it fails to meet COSMIN criteria of relevance, both comprehensiveness, and
414 comprehensibility.

For peer review only - http://bmjopen.bmj.com/site/about/guidelines.xhtml

Improving the quality of MMD-AP's content validity assessment requires qualitative studies involving the target population, including animal shelter workers, lab workers, and veterinary nurses. This could result in changes or the introduction of new items, leading to an updated version of the MMD-AP that would necessitate validation studies.

Limitations and Strengths

Certain limitations must be acknowledged, inherently dependent on the design and execution of this review.

Firstly, COSMIN guidelines provide only a loose directory in cases where reporting of qualitative studies is poor. It gives only general rules for content validity studies, defining what must be reported. It does not provide guidance on how much detail is required to meet the criteria of the COSMIN methodology for assessing content validity [31]. Indeed, COSMIN provides guidelines for an indirect assessment of PROM quality by methodological quality of development and subsequent validation studies of PROMs. Thus, reporting of qualitative studies is one of the main indicators of methodological quality of PROMs [21]. Although COSMIN guidelines precise which methodological steps must be reported, there is no guidance on how much details they should be. Due to this shortcoming, often qualitative studies are insufficiently reported and rated as "doubtful" [31].

Secondly, development studies of PROMs in this review suffer from novelty of COSMIN guidelines. While they started to appear in late 2010s, majority of original development of moral distress scales date to early 2000s [29, 30], before the publication of these guidelines. This may explain the lack of qualitative studies and patient involvement. Yet, while we did see those qualitative methods being introduced for human healthcare professionals, these methods are lacking for veterinary medicine practitioners.

Lastly, moral distress has been coined 40 years ago in the nursing discipline and since then further definitions have been developed, possibly leading to a heterogeneous and complex understanding of moral distress. However, since COSMIN evaluation relies on a degree of subjectivity by reviewers to rate the

For peer review only - http://bmjopen.bmj.com/site/about/guidelines.xhtml

standards of criteria [20], not specific enough definitions and operationalizations may endanger evaluation of PROMs without additional qualitative studies [32]. For example, we encountered both moral stress and moral distress in our first review. Moreover, while we observed that there are many studies focusing on conceptual aspects of moral issues among animal care workers, very few studies used standardized questionnaires or PROM for the moral distress measurements. The use of standardized questionnaires was a criterion for the eligibility in this review. Hence, in the end, we could only identify and evaluate one specific scale for animal care workers. A recent study by Buchbinder et al. (2023) focused on differential assessment of moral stress and moral distress [33]. They discuss that moral stress is linked to systemic overburdening and does not necessarily involve feelings of powerlessness, contrary to moral distress. Moral distress focuses more on specific stressors encountered in clinical settings than on the systemic causes of these stressors in healthcare. Therefore, it is simpler to create questions that reflect these clinical encounters when developing a standardized questionnaire. At the same time, current moral distress scales may suggest that moral distress is an individual pathology, rather than a complex syndrome, due to the lack of attention given to systematic constraints.

To deal with this subjective nature of rating, we relied on the transparent and systemic methodology of COSMIN [20]. The study followed PRISMA guidelines and pre-registered with PROSPERO. This ensures a thorough, transparent, and traceable methodological approach. The adherence to COSMIN guidelines for assessing the content validity of PROMs ensures a rigorous evaluation of the measurement properties of outcome measures. Our evaluation conducted on every version of the scale; and informed by a clearly articulated clinical or research question(s), the methodological quality of PROM design, the development process, findings of the content validity study, and content of the scale itself. By synthesizing robust assessment of COSMIN framework with transparent methodological approach, this study primes the design and standardization of valid MD assessments.

Interpretations

For peer review only - http://bmjopen.bmj.com/site/about/guidelines.xhtml

An accurate assessment of a health problem in terms of incidence or prevalence requests a validated standard for measuring this problem in clinical, occupational, or research settings. It could ideally be a validated diagnostic test, or a consensually approved set of diagnostic criteria, such as those used for quantifying the burden of major depressive disorder or general anxiety disorder in the global burden of diseases [34]. Unless such tests or criteria have been established, PROMs can help estimate the amount of the problem, as it was recently shown in occupational burnout research [35]. Yet, the prerequisite for this is an appropriate choice of the most valid PROM [36, 37]. Compared to occupational burnout, moral distress is a relatively recent construct in occupational psychology, therefore, the research on moral distress could benefit from and capitalize on the methodological development in subjective outcome measurement in the other fields.

For peer review only

Conclusion

This is the first systematic review focused on moral distress measurement in animal care workers. It showed that moral distress is rarely measured using standardized and evidence-based methods. These findings underline the need to develop and thoroughly validate such methods in the context of animal care. We think it is also necessary for the well-being of the animals, as we believe that moral distress has an impact on the animals cared for by the animal caretakers. More generally, methodological improvement and training in psychometry are recommended when developing and validating scales of moral distress and other subjective outcomes.

Figure Legends

Figure 1. Flow-chart of study selection

Declarations

- **Word count:** 5750

For peer review only - http://bmjopen.bmj.com/site/about/guidelines.xhtml

- **Ethics approval and consent to participate**

Not applicable.

- **Consent for publication**

Not applicable.

- **Availability of data and materials**

Data are available online as supplemental material of the present article.

- **Competing interests**

None declared.

- **Funding**

This project has received funding from Swiss National Science Foundation (SNF) for the NRP79 project 407940_206396 “Linking animal and human welfare – refining rodent euthanasia”.

- **Authors' contributions**

Conceptualization, I.G.C.; methodology, N.G., Y.B., and I.G.C.; software, N.G. and Y.B.; validation, N.G., Y.B., I.G.C. and S.H; formal analysis, N.G., and Y.B.; investigation, N.G., Y.B., and I.G.C.; resources, N.G., Y.B., and I.G.C.; data curation, N.G., Y.B., and I.G.C.; writing—original draft preparation, N.B., Y.B., and I.G.C; writing—review and editing, N.G., Y.B., I.G.C. and S.H.; supervision, I.G.C. and S.H ; project administration, I.G.C. and S.H; funding acquisition, I.G.C and S.H. All authors have read and agreed to the published version of the manuscript.

- **Acknowledgements**

For peer review only

503 The authors thank the Unisanté documentarist, Thomas Brauchly, for his precious help in establishing the literature search queries.

For peer review only

For peer review only - http://bmjopen.bmj.com/site/about/guidelines.xhtml

References

1. World Health Organization. WHO guidelines on mental health at work. Geneva: World Health Organization; 2022.
2. Wang H, Abbas KM, Abbasifard M, Abbasi-Kangevari M, Abbastabar H, Abd-Allah F, et al. Global age-sex-specific fertility, mortality, healthy life expectancy (HALE), and population estimates in 204 countries and territories, 1950–2019: a comprehensive demographic analysis for the Global Burden of Disease Study 2019. Lancet. 2020;396(10258):1160-203. doi: 10.1016/S0140-6736(20)30977-6
3. Crane MF, Phillips JK, Karin E. Trait perfectionism strengthens the negative effects of moral stressors occurring in veterinary practice. Aust Vet J. 2015;93(10):354-60. doi: 10.1111/avj.12366.
4. Shoman Y, Rousson V, Bianchi R, Guseva Canu I. Holistic Assessment of Factors Associated with Exhaustion, the Main Symptom of Burnout: A Meta-Analysis of Longitudinal Studies. Int J Environ Res Public Health. 2022;19(20). doi: 10.3390/ijerph192013037.
5. Al-Gobari M, Shoman Y, Blanc S, Canu IG. Point prevalence of burnout in Switzerland: a systematic review and meta-analysis. Swiss Med Wkly. 2022; 152:w30229. doi: 10.4414/smw.2022.w30229.
6. Burdorf A. How to improve intervention research on the psychosocial work environment? Scand J Work Environ Health. 2023;49(5):311-3. doi: 10.5271/sjweh.4107.
7. Zimmermann C, Strohmaier S, Niederkrotenthaler T, Thau K, Schernhammer E. Suicide mortality among physicians, dentists, veterinarians, and pharmacists as well as other high-skilled occupations in Austria from 1986 through 2020. Psychiatry Res. 2023;323:115170. doi:10.1016/j.psychres.2023.115170
8. Moses L, Malowney MJ, Wesley Boyd J. Ethical conflict and moral distress in veterinary practice: A survey of North American veterinarians. J Vet Intern Med. 2018 ;32(6):2115-22. doi: 10.1111/jvim.15315
9. Arbe Montoya AI, Hazel S, Matthew SM, McArthur ML. Moral distress in veterinarians. Vet Rec. 2019;185(20):631. doi: 10.1136/vr.105289.
10. LaFollette MR, Riley MC, Cloutier S, Brady CM, O'Haire ME, Gaskill BN. Laboratory Animal Welfare Meets Human Welfare: A Cross-Sectional Study of Professional Quality of Life, Including Compassion Fatigue, Among Laboratory Animal Personnel. Qual Life Res. 2018; 27(5):1147-57. doi.org/10.3389/fvets.2020.00114
11. Scotney RL, McLaughlin D, Keates HL. A systematic review of the effects of euthanasia and occupational stress in personnel working with animals in animal shelters, veterinary clinics, and biomedical research facilities. J Am Vet Med Assoc. 2015;247(10):1121-30. doi: 10.2460/javma.247.10.1121
12. Grimm H, Biller-Andorno N, Buch T, et al. Advancing the 3Rs: innovation, implementation, ethics and society. Front Vet Sci. 2023;10:1185706. doi:10.3389/fvets.2023.1185706
13. Jameton A. Dilemmas of moral distress: moral responsibility and nursing practice. AWHONNS Clin Issues Perinat Womens Health Nurs. 1993;4(4):542-51.
14. Jameton A, Jackson EM. Nuclear war and nursing ethics. What is the nurse's responsibility? Möbius: J Contin Educ Prof Health Sci. 1984;4(1):75-88. DOI: 10.1002/chp.4760040110

For peer review only - http://bmjopen.bmj.com/site/about/guidelines.xhtml

15. Kogan LR, Rishniw M. Veterinarians and moral distress. J Am Vet Med Assoc. 2023;261(5):1-7. doi: 10.2460/javma.22.12.0598.

16. Reeve C, Rogelberg S, Spitzmueller C, Digiacomo N. The Caring-Killing Paradox: Euthanasia-Related Strain Among Animal-Shelter Workers. J Appl Soc Psychol. 2006;35:119-43. DOI: 10.1111/j.1559-1816.2005.tb02096.x

17. Andrukonis A, Hall NJ, Protopopova A. The Impact of Caring and Killing on Physiological and Psychometric Measures of Stress in Animal Shelter Employees: A Pilot Study. Int J Environ Res Public Health. 2020;17(24). DOI: 10.3390/ijerph17249196.

18. Giannetta N, Villa G, Pennestrì F, Sala R, Mordacci R, Manara DF. Instruments to assess moral distress among healthcare workers: A systematic review of measurement properties. Int J Nurs Stud. 2020; 111:103767. doi: 10.1016/j.ijnurstu.2020.103767.

19. Mokkink LB, de Vet HCW, Prinsen CAC, Patrick DL, Alonso J, Bouter LM, et al. COSMIN Risk of Bias checklist for systematic reviews of Patient-Reported Outcome Measures. Qual Life Res. 2018;27(5):1171-9. DOI: 10.1007/s11136-017-1765-4

20. Terwee CB, Prinsen CAC, Chiarotto A, Westerman MJ, Patrick DL, Alonso J, et al. COSMIN methodology for evaluating the content validity of patient-reported outcome measures: a Delphi study. Qual Life Res. 2018;27(5):1159-70. DOI: 10.1007/s11136 -018-1829-0

21. Mokkink, L.B., Terwee, C.B., Knol, D.L. et al. The COSMIN checklist for evaluating the methodological quality of studies on measurement properties: A clarification of its content. BMC Med Res Methodol 10, 22 (2010). https://doi.org/10.1186/1471-2288-10-22

22. Kingsley C, Patel S. Patient-reported outcome measures and patient-reported experience measures. BJA Educ. 2017;17(4):137-44. DOI: 10.1093/bjaed/mkw060

23. Prinsen CAC, Mokkink LB, Bouter LM, Alonso J, Patrick DL, de Vet HCW, et al. COSMIN guideline for systematic reviews of patient-reported outcome measures. Qual Life Res. 2018;27(5):1147-57. doi: 10.1007/s11136-018-1798-3.

24. Moher D, Liberati A, Tetzlaff J, Altman DG. Preferred reporting items for systematic reviews and meta-analyses: the PRISMA statement. PLoS Med. 2009;6(7):e1000097. doi:10.1371/journal.pmed.1000097.

25. Nosek BA, Ebersole CR, DeHaven AC, Mellor DT. The preregistration revolution. Proc Natl Acad Sci U S A. 2018;115(11):2600-2606. doi:10.1073/pnas.1708274114

26. Ouzzani M, Hammady H, Fedorowicz Z, Elmagarmid A. Rayyan-a web and mobile app for systematic reviews. Syst Rev. 2016;5(1):210. doi.org/10.1186/s13643-016-0384-4

27. Shoman Y, Majery N, Otelea M, Lambreghts C, Guseva Canu I. How to identify the most suitable questionnaires and rating scales for your clinical practice or research? Int J Clin Pract. 2021;75(12): e14895. doi: 10.1111/ijcp.14895

28. Epstein EG, Whitehead PB, Prompahakul C, Thacker LR, Hamric AB. Enhancing Understanding of Moral Distress: The Measure of Moral Distress for Health Care Professionals. AJOB Empir Bioeth. 2019;10(2):113-24. doi: 10.1080/23294515.2019.1586008.

29. Hamric AB. Empirical research on moral distress: issues, challenges, and opportunities. HEC Forum. 2012;24(1):39-49. doi: 10.1007/s10730-012-9177-x.

30. Corley MC, Elswick RK, Gorman M, Clor T. Development and evaluation of a moral distress scale. J Adv Nurs. 2001;33(2):250-6. doi: 10.1046/j.1365-2648.2001.01658.x.

For peer review only - http://bmjopen.bmj.com/site/about/guidelines.xhtml

590 31. Rothmund, M., Meryk, A., Rumpold, G. et al. A critical evaluation of the content validity of
591 patient-reported outcome measures assessing health-related quality of life in children
592 with cancer: a systematic review. J Patient Rep Outcomes 7, 2 (2023).
593 DOI: https://doi.org/10.1186/s41687-023-00540-8
594 32. van Andel EM, Koopmann BDM, Crouwel F, Noomen CG, de Boer NKH, van Asseldonk DP,
595 Mokkink LBM. Systematic Review of Development and Content Validity of Patient
596 reported Outcome Measures in Inflammatory Bowel Disease: Do We Measure What
597 We Measure?. J Crohn's Colitis. 2020;14(9):1299–1315.
598 https://doi.org/10.1093/ecco-jcc/jjaa057
599 33. Buchbinder M, Browne A, Berlinger N, Jenkins T, Buchbinder L. Moral Stress and Moral
600 Distress: Confronting Challenges in Healthcare Systems under Pressure. Am J Bioeth.
601 2023. doi:10.1080/15265161.2023.2224270
602 34. Santomauro DF, Mantilla Herrera AM, Shadid J, Zheng P, Ashbaugh C, Pigott DM, et al. Global
603 prevalence and burden of depressive and anxiety disorders in 204 countries and
604 territories in 2020 due to the COVID-19 pandemic. Lancet. 2021;398(10312):1700-12.
605 DOI: https://doi.org/10.1016/S0140-6736(21)02143-7
606 35. Guseva Canu I, Marca SC, Dell'Oro F, Balázs Á, Bergamaschi E, Besse C, et al. Harmonized
607 definition of occupational burnout: A systematic review, semantic analysis, and Delphi
608 consensus in 29 countries. Scand J Work Environ Health. 2021;(2):95-107. doi:
609 10.5271/sjweh.3935.
610 36. Guseva Canu I, Mesot O, Györkös C, Mediouni Z, Mehlum IS, Bugge MD. Burnout syndrome in
611 Europe: towards a harmonized approach in occupational health practice and research. Ind
612 Health. 2019;57(6):745-52. doi: 10.2486/indhealth.2018-0159.
613 37. Shoman Y, Marca S, Bianchi R, Godderis L, Molen HF, Guseva Canu I. Psychometric properties
614 of burnout measures: A systematic review. Epidemiol Psychiatr Sci. 2021;30:e8. doi:
615 10.1017/S2045796020001134.

For peer review only - http://bmjopen.bmj.com/site/about/guidelines.xhtml

Table 1. Quality of the PROM development

**PROM**	**MMD-HP**	**MMD-AP**
First author, year	Esptein et al., 2019	Kogan et al., 2023
Language in which the PROM was developed	English	English
**PROM design**		
Clear construct	V	V
Clear origin of construct	V	V
Clear target population for which the PROM was developed	V	V
Clear context of use	V	V
PROM developed in sample representing the target population	V	V
Concept elicitation	A	D
Total PROM design	***A***	***D***
Cognitive interview study performed in sample representing the target population	A	V
Comprehensibility	D	D
Total Cognitive interview study	***D***	***D***
**Total PROM development**	**D**	**D**

Note*: V: very good, A: adequate, D: doubtful*

For peer review only - http://bmjopen.bmj.com/site/about/guidelines.xhtml

Table 2. Systematic results for the MMD-HP and MMD-AP PROMs for the criteria content validity

PROM	Development study	Rating of reviewers	Overall rating + / - / ?	Quality of evidence
**MMD-HP**				
Relevance	+	±	+	**Low**
Comprehensiveness	+	+	+	**Low**
Comprehensibility	+	+	+	**Low**
**Content validity rating**	+	+	+	**Low**
**MMD-AP**				
Relevance	±	+	±	**Low**
Comprehensiveness	-	+	±	**Low**
Comprehensibility	-	+	±	**Low**
**Content validity rating**	±	+	±	**Low**

Note: +*: sufficient, -: insufficient,* ±: *inconsistent*

For peer review only - http://bmjopen.bmj.com/site/about/guidelines.xhtml

Table 3. Systematic results for the overall rating of MMD-HP and MMD-AP PROMs and their quality of evidence

**PROM**	**MMD-HP**		**MMD-AP**	
**COSMIN psychometric properties for content validity**	**Overall rating + / - / ?**	**Quality of evidence High, moderate, low, very low**	**Overall rating + / - / ?**	**Quality of evidence High, moderate, low, very low**
Relevance	+	**Low**	±	**Low**
Comprehensiveness	+	**Low**	**-**	**Low**
Comprehensibility	+	**Low**	**-**	**Low**

Note: +*: sufficient, -: insufficient,* ±: *inconsistent*

For peer review only

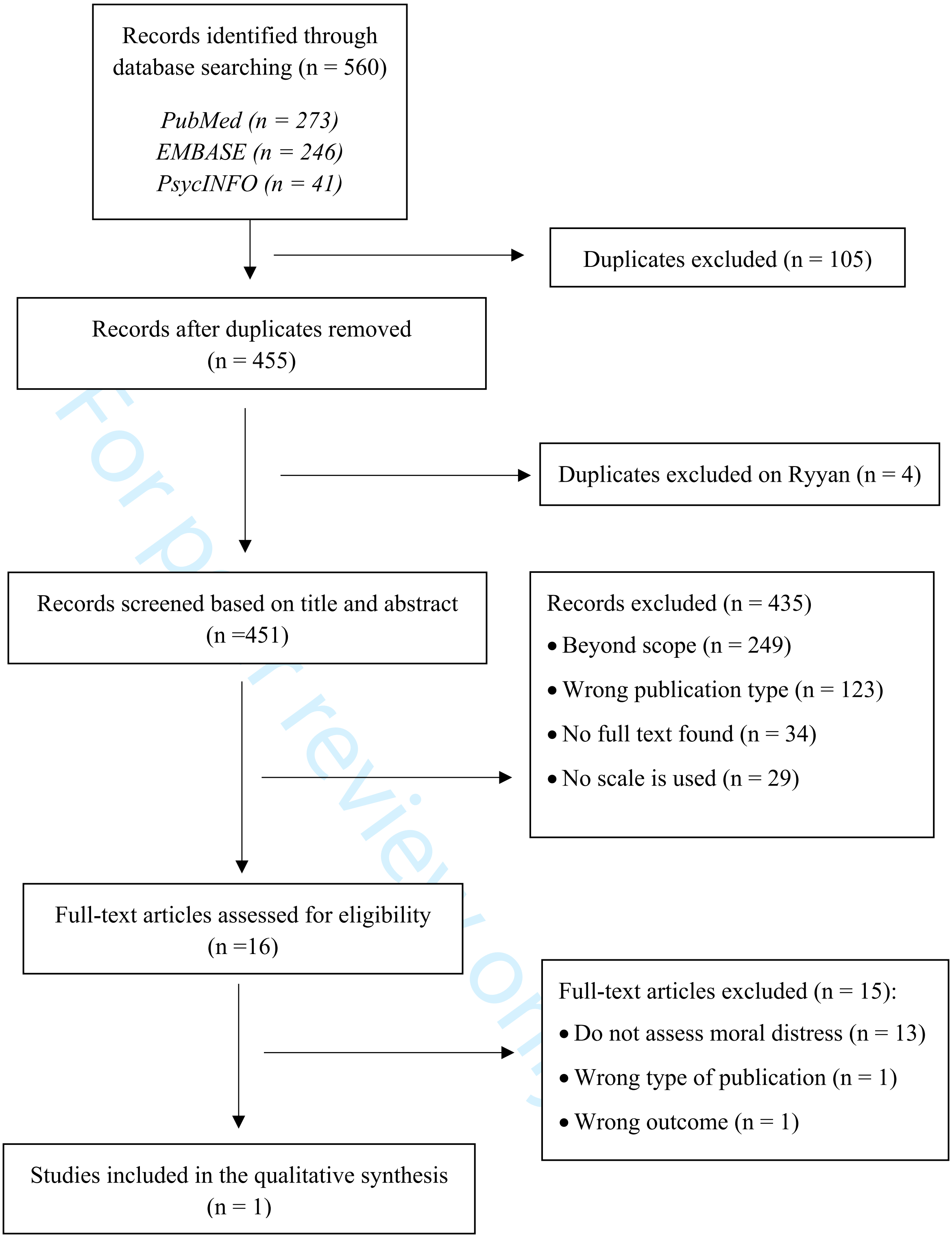

Figure 1: Flow-chart

PRISMA 2020 Checklist

Section and Topic	Item #	Checklist item	Location where item is reported
**TITLE**			
Title	1	Identify the report as a systematic review.	1
**ABSTRACT**			
Abstract	2	See the PRISMA 2020 for Abstracts checklist.	21
**INTRODUCTION**			
Rationale	3	Describe the rationale for the review in the context of existing knowledge.	68
Objectives	4	Provide an explicit statement of the objective(s) or question(s) the review addresses.	118
**METHODS**			
Eligibility criteria	5	Specify the inclusion and exclusion criteria for the review and how studies were grouped for the syntheses.	141
Information sources	6	Specify all databases, registers, websites, organisations, reference lists and other sources searched or consulted to identify studies. Specify the date when each source was last searched or consulted.	147
Search strategy	7	Present the full search strategies for all databases, registers and websites, including any filters and limits used.	Supplemental materials
Selection process	8	Specify the methods used to decide whether a study met the inclusion criteria of the review, including how many reviewers screened each record and each report retrieved, whether they worked independently, and if applicable, details of automation tools used in the process.	155
Data collection process	9	Specify the methods used to collect data from reports, including how many reviewers collected data from each report, whether they worked independently, any processes for obtaining or confirming data from study investigators, and if applicable, details of automation tools used in the process.	158
Data items	10a	List and define all outcomes for which data were sought. Specify whether all results that were compatible with each outcome domain in each study were sought (e.g. for all measures, time points, analyses), and if not, the methods used to decide which results to collect.	189
	10b	List and define all other variables for which data were sought (e.g. participant and intervention characteristics, funding sources). Describe any assumptions made about any missing or unclear information.	Not applicable
Study risk of bias assessment	11	Specify the methods used to assess risk of bias in the included studies, including details of the tool(s) used, how many reviewers assessed each study and whether they worked independently, and if applicable, details of automation tools used in the process.	184
Effect measures	12	Specify for each outcome the effect measure(s) (e.g. risk ratio, mean difference) used in the synthesis or presentation of results.	Tables 1,2 and 3
Synthesis methods	13a	Describe the processes used to decide which studies were eligible for each synthesis (e.g. tabulating the study intervention characteristics and comparing against the planned groups for each synthesis (item #5)).	192
	13b	Describe any methods required to prepare the data for presentation or synthesis, such as handling of missing summary statistics, or data conversions.	Not applicable
	13c	Describe any methods used to tabulate or visually display results of individual studies and syntheses.	Not applicable
	13d	Describe any methods used to synthesize results and provide a rationale for the choice(s). If meta-analysis was performed, describe the model(s), method(s) to identify the presence and extent of statistical heterogeneity, and software package(s) used.	Not applicable
	13e	Describe any methods used to explore possible causes of heterogeneity among study results (e.g. subgroup analysis, meta-regression).	Not applicable
	13f	Describe any sensitivity analyses conducted to assess robustness of the synthesized results.	Not

For peer review only - http://bmjopen.bmj.com/site/about/guidelines.xhtml

PRISMA 2020 Checklist

Section and Topic	Item #	Checklist item	Location where item is reported
			applicable
Reporting bias assessment	14	Describe any methods used to assess risk of bias due to missing results in a synthesis (arising from reporting biases).	Not applicable
Certainty assessment	15	Describe any methods used to assess certainty (or confidence) in the body of evidence for an outcome.	Not applicable
**RESULTS**			
Study selection	16a	Describe the results of the search and selection process, from the number of records identified in the search to the number of studies included in the review, ideally using a flow diagram.	200
	16b	Cite studies that might appear to meet the inclusion criteria, but which were excluded, and explain why they were excluded.	204
Study characteristics	17	Cite each included study and present its characteristics.	206
Risk of bias in studies	18	Present assessments of risk of bias for each included study.	346/ Table 3
Results of individual studies	19	For all outcomes, present, for each study: (a) summary statistics for each group (where appropriate) and (b) an effect estimate and its precision (e.g. confidence/credible interval), ideally using structured tables or plots.	Tables 1,2 and 3
Results of syntheses	20a	For each synthesis, briefly summarise the characteristics and risk of bias among contributing studies.	331 /Table 2
	20b	Present results of all statistical syntheses conducted. If meta-analysis was done, present for each the summary estimate and its precision (e.g. confidence/credible interval) and measures of statistical heterogeneity. If comparing groups, describe the direction of the effect.	Not applicable
	20c	Present results of all investigations of possible causes of heterogeneity among study results.	Not applicable
	20d	Present results of all sensitivity analyses conducted to assess the robustness of the synthesized results.	Not applicable
Reporting biases	21	Present assessments of risk of bias due to missing results (arising from reporting biases) for each synthesis assessed.	Not applicable
Certainty of evidence	22	Present assessments of certainty (or confidence) in the body of evidence for each outcome assessed.	Not applicable
**DISCUSSION**			
Discussion	23a	Provide a general interpretation of the results in the context of other evidence.	456
	23b	Discuss any limitations of the evidence included in the review.	414
	23c	Discuss any limitations of the review processes used.	Not applicable
	23d	Discuss implications of the results for practice, policy, and future research.	470
**OTHER INFORMATION**			
Registration and protocol	24a	Provide registration information for the review, including register name and registration number, or state that the review was not registered.	51
	24b	Indicate where the review protocol can be accessed, or state that a protocol was not prepared.	51
	24c	Describe and explain any amendments to information provided at registration or in the protocol.	Not

For peer review only - http://bmjopen.bmj.com/site/about/guidelines.xhtml

PRISMA 2020 Checklist

Section and Topic	Item #	Checklist item	Location where item is reported
			applicable
Support	25	Describe sources of financial or non-financial support for the review, and the role of the funders or sponsors in the review.	52
Competing interests	26	Declare any competing interests of review authors.	486
Availability of data, code and other materials	27	Report which of the following are publicly available and where they can be found: template data collection forms; data extracted from included studies; data used for all analyses; analytic code; any other materials used in the review.	Supplemental materials

From: Page MJ, McKenzie JE, Bossuyt PM, Boutron I, Hoffmann TC, Mulrow CD, et al. The PRISMA 2020 statement: an updated guideline for reporting systematic reviews. BMJ 2021;372:n71. doi: 10.1136/bmj.n71

For more information, visit: http://www.prisma-statement.org/

For peer review only
